# Iron‐Catalyzed Laser‐Induced Graphitization Enabling Current Collector‐Free Electrodes With Spatially Tunable Iron/Iron Oxide Phases

**DOI:** 10.1002/adma.202508812

**Published:** 2025-08-01

**Authors:** Christopher H. Dreimol, Jesper Edberg, Ronny Kürsteiner, Maximilian Ritter, Sophie Koch, Annapaola Parrilli, Robert O. Kindler, Robert Brooke, Susanna Tinello, Sandro Stucki, Simon Bryner, Gerd Simons, Guido Panzarasa, Ingo Burgert

**Affiliations:** ^1^ Wood Materials Science Institute for Building Materials ETH Zürich Zürich 8093 Switzerland; ^2^ Cellulose & Wood Materials Laboratory Empa Dübendorf 8600 Switzerland; ^3^ RISE Research Institutes of Sweden Digital Systems Smart Hardware Bio‐ and Organic Electronics Norrköping 60233 Sweden; ^4^ Center for X‐ray Analytics Empa – Swiss Federal Laboratories for Materials Science and Technology Dübendorf 8600 Switzerland; ^5^ Laboratory for Multifunctional Materials Department of Materials ETH Zürich Zürich 8093 Switzerland; ^6^ Institute for Sensors and Electronics School of Engineering FHNW Windisch 5210 Switzerland

**Keywords:** core‐shell nanoparticles, hybrid carbon‐iron electrodes, multilayer electrode designs, supercapacitors, sustainable energy storage materials

## Abstract

Iron‐catalyzed laser‐induced graphitization (IC‐LIG) represents an eco‐efficient alternative to traditional carbon electrode manufacturing. Combining a bio‐based tannic acid–iron precursor ink with CO_2_ laser treatment results in sheet resistance of 23.59 ± 1.2Ω □^−1^ on renewable substrates. Varying the tannic‐acid‐to‐iron ratio (TA:Fe), the rheology of the precursor ink can be tuned, enabling versatile application techniques, including spray coating, screen printing, and direct‐ink‐writing (DIW). Subsequent laser‐treatment enables the formation of functional IC‐LIG electrodes for all application methods, while even thick DIW‐printed layers (260 µm) result in complex, conductive electrode patterns. Laser post‐treatment expands design possibilities by locally tuning iron phases, such as converting γ‐iron to magnetite. The unidirectional laser‐treatment results in a layered arrangement, forming a multilayer electrode with a highly graphitized top layer serving as a current collector substitute, and an underlying composite of iron‐rich nanoparticles embedded in a porous graphitic foam, acting as a hybrid electrode. Electrochemical analysis reveals double‐layer capacitor behavior at low TA:Fe ratios, while higher ratios demonstrate increased redox activity and pseudo‐capacitive characteristics. Achieving stable capacities of 15 mF cm^−2^ with a 1 M NaCl electrolyte over 5000 cycles underscores the potential of IC‐LIG electrodes as a sustainable solution for advanced energy storage devices and beyond.

## Graphical abstract

Iron‐catalyzed laser‐induced graphitization (IC‐LIG) enables the eco‐efficient fabrication of current collector‐free carbon electrodes on renewable substrates, outperforming traditional methods. Tunable ink rheology supports spray coating, screen printing, and DIW of complex patterns, while laser post‐treatment allows spatial control of iron phases. Graphitic structures support redox‐active phases, enabling stable performance, offering a sustainable platform for future electrochemical devices and beyond.

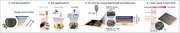

## 1 Introduction

The pursuit of increasingly efficient energy storage devices has resulted in the continuous development of high‐performance carbon‐based electrode materials.^[^ ^1^, ^2^, ^3^, ^4^ ^]^ Especially the hybridization of carbon (nano) materials with transition‐metal oxides and carbides (e.g., MXenes), such as the decoration of graphite sheets with Fe_3_O_4_^[^ ^5^, ^6^, ^7^, ^8^, ^9^ ^]^ or graphite–MXenes composites,^[^ ^10^ ^]^ is anticipated to enable the roll‐out of next‐generation, current collector‐free^[^ ^11^, ^12^, ^13^ ^]^ energy storage devices.^[^ ^14^ ^]^ This could be achieved through the synergistic combination of functionalities, such as high electronic conductivity from graphite and high redox activity from (transition) metal oxides and carbides.^[^ ^15^ ^]^ However, these hybrid electrodes present challenges related to cost, manufacturability, and recyclability, including complex processing methods, hazardous chemicals, and difficult‐to‐recycle waste streams.^[^ ^16^ ^]^ To meet the growing demand for carbon‐based and hybrid electrodes in future markets and to help reducing the generation of unmanageable amounts of electronic waste, the current research and industry focus is shifting toward developing competitive, eco‐efficient processes based on renewable and sustainable materials.^[^ ^1^, ^17^, ^18^, ^19^ ^]^

Laser‐induced graphitization (LIG) represents an emerging technology^[^ ^20^ ^]^ that enables the conversion of polymeric^[^ ^5^, ^21^, ^22^, ^23^, ^24^, ^25^, ^26^ ^]^ and organic precursors^[^ ^27^, ^28^, ^29^, ^30^, ^31^, ^32^, ^33^, ^34^, ^35^ ^]^ directly into graphitic electrodes. The laser is employed as a localized, surface‐selective heat source for the graphitization process. By varying the process parameters, such as laser writing speed, power, and defocus, the LIG electrode structure can be tailored, including porosity and surface chemistry,^[^ ^36^, ^37^ ^]^ alongside its electrical conductivity.^[^ ^23^ ^]^ Exploiting renewable, biomass‐derived precursors for LIG is particularly noteworthy in the context of energy storage materials, given the environmental impact associated with the harvesting, production, and processing of conventional graphite electrodes.^[^ ^14^ ^]^ It is further worth emphasizing that conventional laser processes are already being considered for integration into the industrial processing of electrodes, for example, for cutting, patterning, and drying.^[^ ^38^, ^39^, ^40^ ^]^ Despite recent encouraging advances in the implementation of LIG electrodes in energy storage devices, including electrochemical double‐layer capacitors (EDLC),^[^ ^27^, ^35^, ^41^ ^]^ the potential for employing redox‐active materials remains yet to be fully realized.^[^ ^42^ ^]^ A major hindrance is the complex, multi‐step manufacturing processes, which can involve multiple laser treatments, post‐modification with metal salt solutions, and electrodeposition of transition metals.^[^ ^43^, ^44^ ^]^

Here, we propose an eco‐efficient, direct fabrication of a current collector‐free, hybrid carbon‐iron (oxide) electrode for sustainable energy storage devices. We build upon our recently introduced iron‐catalyzed laser‐induced graphitization (IC‐LIG) approach,^[^ ^32^, ^45^ ^]^ which combines the catalytic graphitization behavior of an aqueous precursor ink (containing an iron(III)‐tannic acid coordination complex) with a commercial CO_2_ laser treatment.

It has been documented that the complexation behavior between TA and iron ions depends on their molar ratio,^[^ ^46^, ^47^ ^]^ which allows for the control of film thickness and nanoparticle size.^[^ ^48^ ^]^ In this study, the tannic acid‐to‐iron ratio (TA:Fe), ranging from 1:0.5 to 1:4, is modified to assess the processability of the precursor inks as well as the structure–property relationship of IC‐LIG electrodes, where Fe refers to the molar amount of Fe^3^⁺ ions supplied by ammonium iron(III) citrate. By varying the quantity of iron(III) ions present in the precursor ink, the composition of the iron‐tannic acid complex is modified, which in turn affects the rheological properties of the ink. Since rheological properties influence how the ink can be applied, tailoring them is crucial for enabling diverse application techniques.^[^ ^49^ ^]^ Thus, we aimed to optimize the ink's rheology to ensure compatibility with various deposition methods, streamlining the IC‐LIG process for potential industrial scale‐up. To this end, we explore the feasibility of applying iron‐tannic acid precursor inks via spray coating, semi‐automated screen printing, and direct ink writing (DIW). Subsequent laser treatment enabled catalytic graphitization via IC‐LIG across all printed structures. A key highlight is the excellent structural integrity and stability of thick (40 to 260 µm) DIW‐printed electrodes on wood pulp board (WPB) and plastic substrates, allowing the fabrication of complex electrode patterns. Furthermore, laser post‐treatments were employed as spatially confined heat sources to investigate their influence on iron phase transformations. Defocused, low‐energy laser treatment resulted in broad heat‐affected zones, enabling a spatial phase transition from γ‐Fe to predominantly magnetite (Fe_3_O_4_), while focused high‐energy laser exposure induced local ablation, thereby facilitating electrode patterning.

IC‐LIG electrodes derived from three different TA:Fe ratios (1:1, 1:2, and 1:3) were selected for in‐depth structural analysis to investigate the catalytic graphitization and structural evolution. Furthermore, laser post‐treatments were performed as an additional and spatially confined heat source to study their effects on the iron phases. A layered structure with a highly graphitized top layer was observed for all investigated TA:Fe ratios. This structural feature is exploited to introduce a functional separation between the layers, leading to the development of a novel multilayer electrode design. The top layer serves as an alternative to a metal current collector, while the subsequent layers—comprising iron‐rich nanoparticles embedded within a porous graphitic foam—function as a hybrid electrode. Since the TA:Fe ratio directly influences the amount of redox‐active iron species within the IC‐LIG electrode, we investigated its electrochemical performance, suggesting the possibility of tuning IC‐LIG electrodes for different electrochemical applications. Thus, IC‐LIG electrodes with low iron content and a high surface area are envisioned for EDLCs, while those with a higher concentration of redox‐active iron species are suited for pseudo‐capacitor applications, benefiting from their high redox potential.

Unlike conventional multi‐step electrode fabrication,^[^ ^39^, ^50^ ^]^ the IC‐LIG approach enables an integrated process in which both the current collector and the electrochemically active layer are formed from the same bio‐based precursor via a single laser‐induced graphitization step. This streamlined strategy eliminates the need for electrode binders, conductive additives (e.g., graphite and carbon black), and solvent along with its associated energy‐intensive drying processes.^[^ ^39^ ^]^ It also removes the need for separate metal current collectors, thereby reducing material usage, manufacturing costs, and total device weight.^[^ ^18^ ^]^ Together, these advantages position IC‐LIG as a modular and scalable platform, where ink formulation, laser parameters, and substrate selection can be tailored to meet the requirements of a wide range of applications, including electrochemical energy storage, harvesting, and sensing applications—where low cost, high performance, and sustainability are essential.

## 2 Results and Discussion

### 2.1 Ink Rheology and Resulting Application Methods

**Figure 1:**
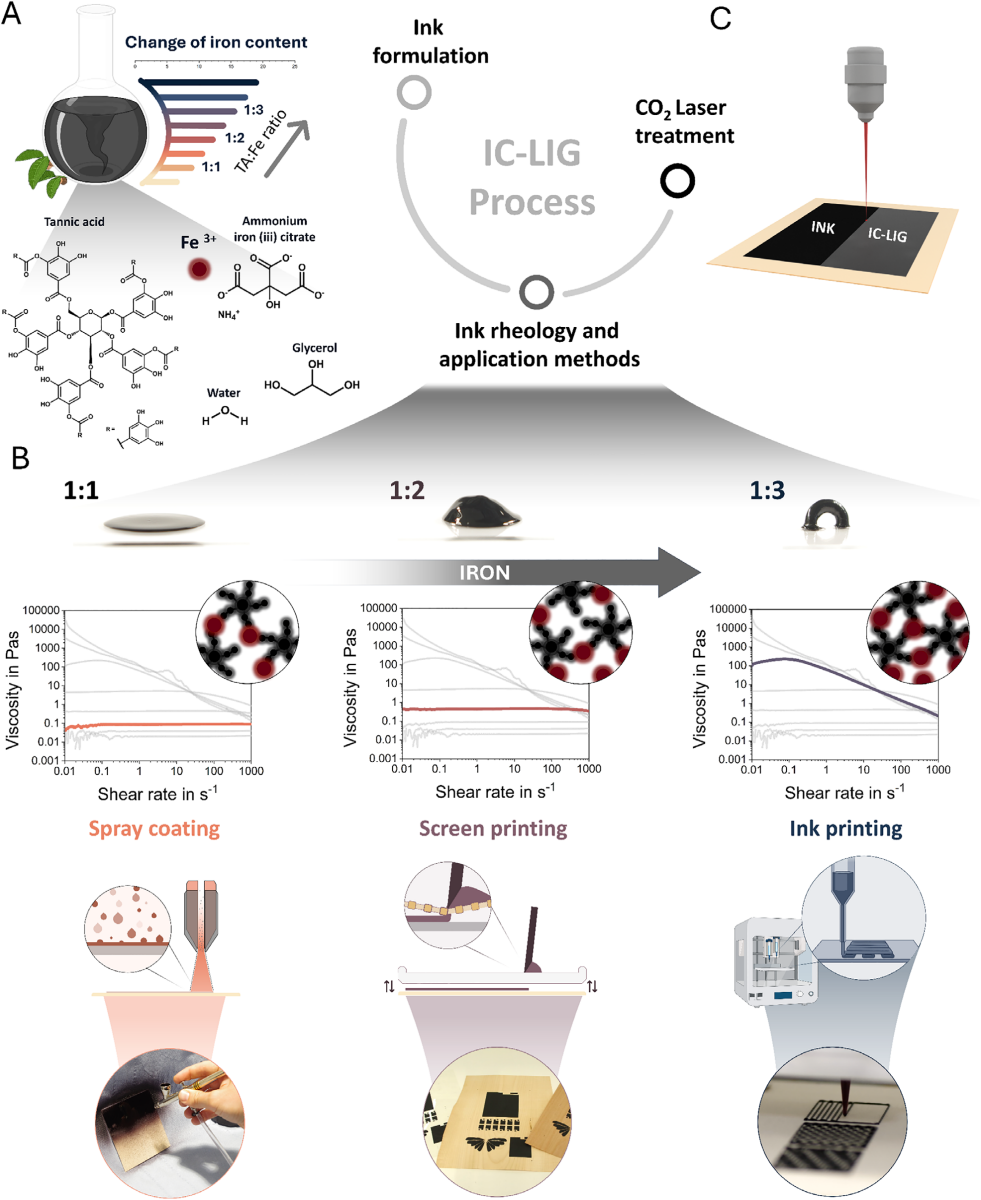
A) Schematic overview of the IC‐LIG process, including ink formulation with corresponding tannic acid (TA) to Fe^3^⁺ ion ratios (provided via ammonium iron(III) citrate). B) Influence of the TA:Fe ratio (1:1, 1:2, 1:3) on ink viscosity and supramolecular coordination. A photograph of three formulated inks on glass substrates visually demonstrates increasing viscosity with higher Fe^3^⁺ content. The circular insets schematically illustrate the increasing density of Fe^3^⁺–TA coordination complexes. Ruby‐colored circles consistently represent Fe^3^⁺ ions; only their number changes to reflect varying coordination degrees. Schematic illustrations and corresponding photographs of the application methods including spray coating, screen printing, and DIW, are also shown. C) A CO_2_ laser treatment then converts the ink‐coated substrates into conductive IC‐LIG electrodes through localized graphitization.

**Figure** **1** provides a schematic representation of the individual steps of the IC‐LIG approach, starting with the ink formulation. In this initial step, the components are thoroughly mixed, including water as solvent, glycerol as plasticizer, ammonium iron(III) citrate as catalyst, and tannic acid as carbon source. To understand the influence of the iron amount on the processing properties and electrochemical performance of the resulting laser‐treated IC‐LIG electrodes, inks were prepared with a range of different TA:Fe ratios. By adjusting the amount of ammonium iron(III) citrate, nine formulations were prepared with the Ta:Fe ratio ranging from 1:0.25 to 1:4 in 0.5‐step increments (Table S1, Supporting Information). To comprehensively evaluate the possibility of different application techniques, we initially tested the rheological properties of all ink formulations (Figure S1, Supporting Information). Subsequently, the ratios of 1:1, 1:2, and 1:3 were selected for spray coating, semi‐automated screen printing, and DIW, respectively.

Significant changes in viscosity were observed upon ink preparation, as varying the TA:Fe ratios directly affected the rheological characteristics of the ink formulations. These differences in rheological behavior can be attributed to the iron‐tannic acid complex. As schematically illustrated in Figure 1B, the black star‐like molecules represent the polyphenolic structure of TA, which consists of five digalloyl ester groups covalently bound to a central glucose core.^[^ ^51^ ^]^ The galloyl groups are capable of forming coordination bonds with the trivalent iron cation Fe^3+^, as represented by the colored spheres. Here, the TA:Fe ratio plays a crucial role in the formation of complexes in solution.^[^ ^52^, ^53^ ^]^ As the concentration of Fe^3+^ ions increases, the probability of forming tris‐type complexes become more prevalent.^[^ ^46^ ^]^ Accordingly, the disparate rheological behavior, can be attributed to the coordination bonds between the iron ions and the tannic acid molecules.^[^ ^54^ ^]^ Low amounts of iron result in low‐viscous, Newtonian inks, while increasing the iron content further leads to shear‐thinning, self‐supporting pastes, as shown in Figure 1B. To illustrate this, a drop of ink with a TA:Fe ratio of 1:1 was deposited on a glass slide, where it spread instantaneously and homogeneously. In contrast, a TA:Fe ratio of 1:3 allowed the formation of a free‐standing arched bead (Figure 1B).

The low‐viscosity inks facilitate their application with spray coating, a fast, efficient, and large‐area coating method. During spray coating, the ink is mixed with a compressed air stream, atomized into fine droplets, and released as a fine spray, as illustrated in Figure 1B. The requisite viscosity for spray coating is between 0.01 and 0.1 Pa s,^[^ ^49^ ^]^ which is achieved by the TA:Fe ratio between 1:0.5 and 1:1 (Figure 1B). To demonstrate the potential of spray coating, an airbrush was employed to coat wood veneers. The coating was found to be homogeneous and thin, with film thickness increasing upon the application of multiple layers (Figure S2, Supporting Information; ≈50 µm after applying three consecutive layers), as rapid drying of each sprayed ink layer was observed (<60 s at 20 °C).

The viscosity requirements for screen printing are different. A viscosity between 0.5 and 5 Pa s, is necessary to facilitate the transfer of ink through the screen (mesh) using a flood bar, as illustrated in the schematic in Figure 1B. The patterned screen determines the features transferred to the substrate. The viscous behavior of the ink prevents it from spreading, thereby allowing for self‐supporting, detailed features until the ink fully dries. In screen printing, the film thickness is typically within the range of 5 to 100 µm.^[^ ^49^ ^]^ To demonstrate the potential of screen printing, we employed a semi‐automated screen‐printing apparatus and an ink formulation with a TA:Fe ratio of 1:2 that fulfills the rheological requirements (Figure 1B, Figure S3, Supporting Information). It is noteworthy that not only diverse substrates (wood, WPB, and plastic foils) and multiple, consecutive layers are feasible, but the ink also exhibits minimal spreading, and precise details (Figure S3, Supporting Information), enabling coating on substrates with considerable surface roughness, as observed for wood veneers.

Eventually, high‐viscosity inks, with a viscosity greater than 100 Pa·s such as those with a TA:Fe ratio of 1:3, can be used for extrusion‐based 3D printing via DIW by means of a bioplotter (Figure 1B, Figure S4, Movie S1, Supporting Information). As illustrated in the schematic representations, the ink is filled into a cartridge and extruded through a nozzle utilizing pneumatic pressure. By modulating applied pressure, printing speed, and line pitch, it is possible to precisely control feature size and deposit homogeneous, though thick, coatings or intricate patterns. Here, formulations with TA:Fe ratios as high as 1:3 result in favorable shear‐thinning behavior, preventing nozzle clogging during DIW while allowing the stable formation of free‐standing geometries (Figure 1B).

### 2.2 Influence of the TA:Fe Ratio on the Structural Evolution of IC‐LIG

In order to compare structural differences derived from the TA:Fe ratios, aWPB produced from mechanical pulp was selected as a model substrate. The WPB has a similar chemical composition to native wood, yet without the structural inhomogeneity observed on a native wood surface. The film thickness was adjusted to ≈100 microns for all inks by brush coating as a universal application technique, allowing all three inks to be applied consistently and ensuring comparability.

The IC‐LIG approach proceeds with the laser graphitization of the ink‐coated WPB samples, which produces a graphitic foam, thus constituting the IC‐LIG electrode. A recently published multi‐scale analysis of an IC‐LIG electrode, derived from an ink with a TA:Fe ratio of 1:2 on a WPB substrate, showed that the unidirectional laser treatment resulted in different heat zones, both in‐plane and across the sample thickness, leading to the formation of a layered structure. In addition, the different heat zones influenced the size and phase of the catalytic nanoparticles and, consequently, the degree of graphitization of the carbon precursor.^[^ ^45^ ^]^ Building on these initial findings, a similar multi‐scale analysis was employed to investigate the structural differences resulting from the varying TA:Fe ratios in the precursor inks. Three IC‐LIG electrodes, made with inks containing TA:Fe in ratios of 1:1, 1:2, and 1:3, were selected for a detailed structural investigation. It is important to note that TA:Fe ratios below 1:0.5 showed high ablation during laser graphitization (under the specific lasing parameters used in this study, as shown in Figure S5, Supporting Information), resulting in non‐conductive surfaces, thus marking the lower threshold for possible TA:Fe ratios.

**Figure 2:**
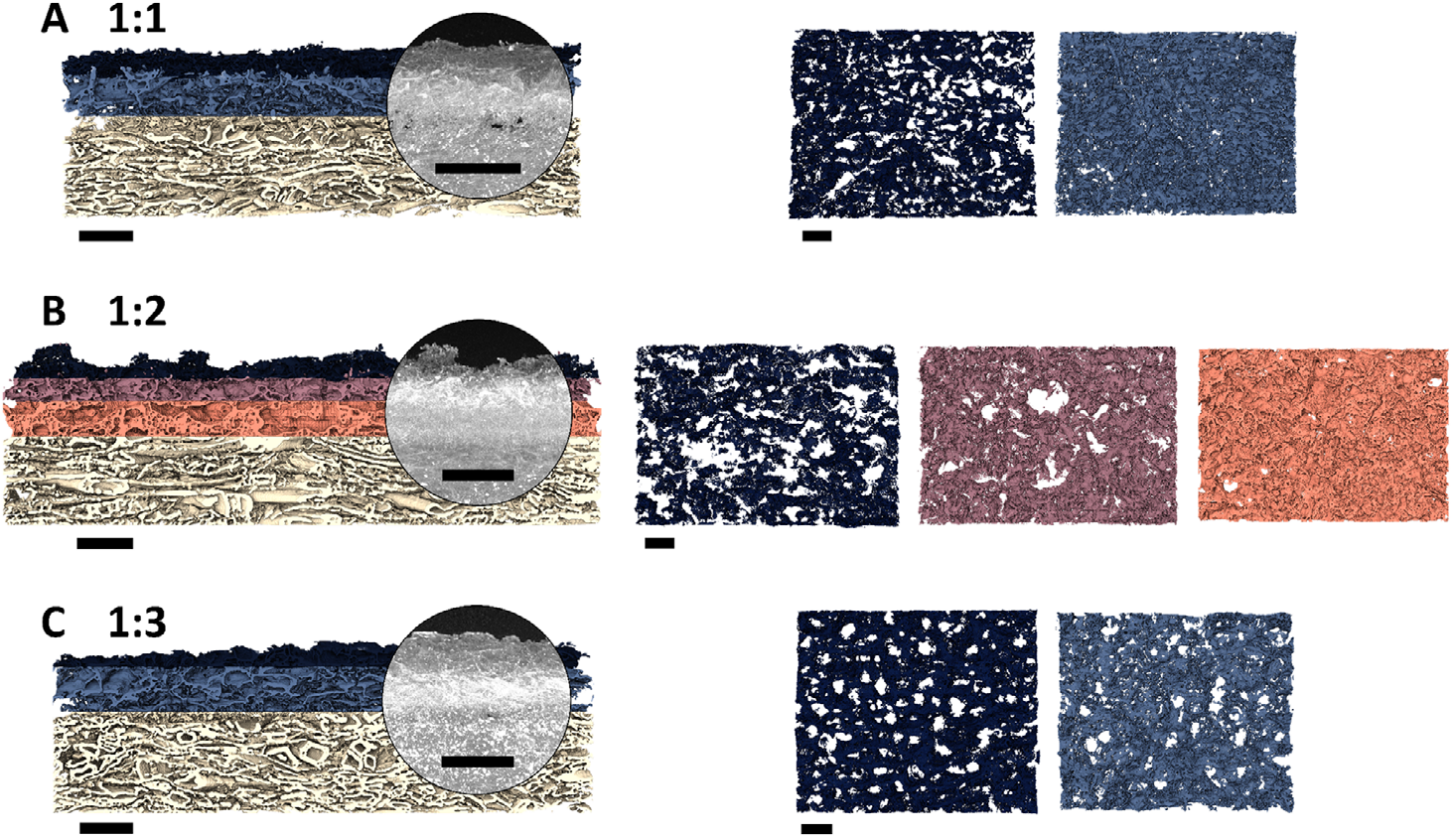
3D reconstruction of single nano‐CT images of cross‐sectional samples with corresponding maximum intensity projection A, B, C) together with their separated layers in top view perspective, highlighting the porosity changes between the three different TA:Fe ratios (1:1, 1:2, 1:3). Scale bars are 100 µm.

The 3D image reconstructions obtained from the nano‐CT image acquisition (**Figure** **2**) allowed comparing structural differences on a mesostructural level. Here, we observed differences in intensity within the maximum intensity projection (MIP, 2D projection as image overlay of single nano‐CT image slices) across the sample thickness (Figure 2). The observed differences in intensity were attributed to variations in material composition, thereby enabling their separation within the 3D nano‐CT image reconstructions, resulting in an overall layered arrangement. For the sample with a TA:Fe ratio of 1:2, a three‐layer structure was observed, as described in a previously published study (Figure 2B). In contrast, the 1:1 and 1:3 samples are distinguished by a two‐layer structure (Figure 2A–C). Here, in addition to the top layer, a more intense bottom layer can be observed. However, the bottom layer's intensity is significantly higher in the sample with a TA:Fe ratio of 1:3, likely due to its higher amount of iron‐rich (nano)particles, which exhibit high X‐ray attenuation. The 3D reconstruction allowed further comparison of the porosity for each individual layer. Sample 1:2 clearly shows a gradual transition in porosity, with the top layer having a high porosity of 93%, the middle layer 77%, and the bottom layer a lower porosity of 67%. In contrast, the porosities between the top and bottom layers of samples 1:1 and 1:3 are similar, with the porosity of the top layer being 86% for both samples, while the bottom layer has a porosity of 87% and 84% for samples 1:1 and 1:3, respectively. It is noteworthy that the top layer of sample 1:1 is characterized by large pores and a visibly smoother surface compared to samples 1:2 and 1:3, where the top layer is irregular and rough.

**Figure 3:**
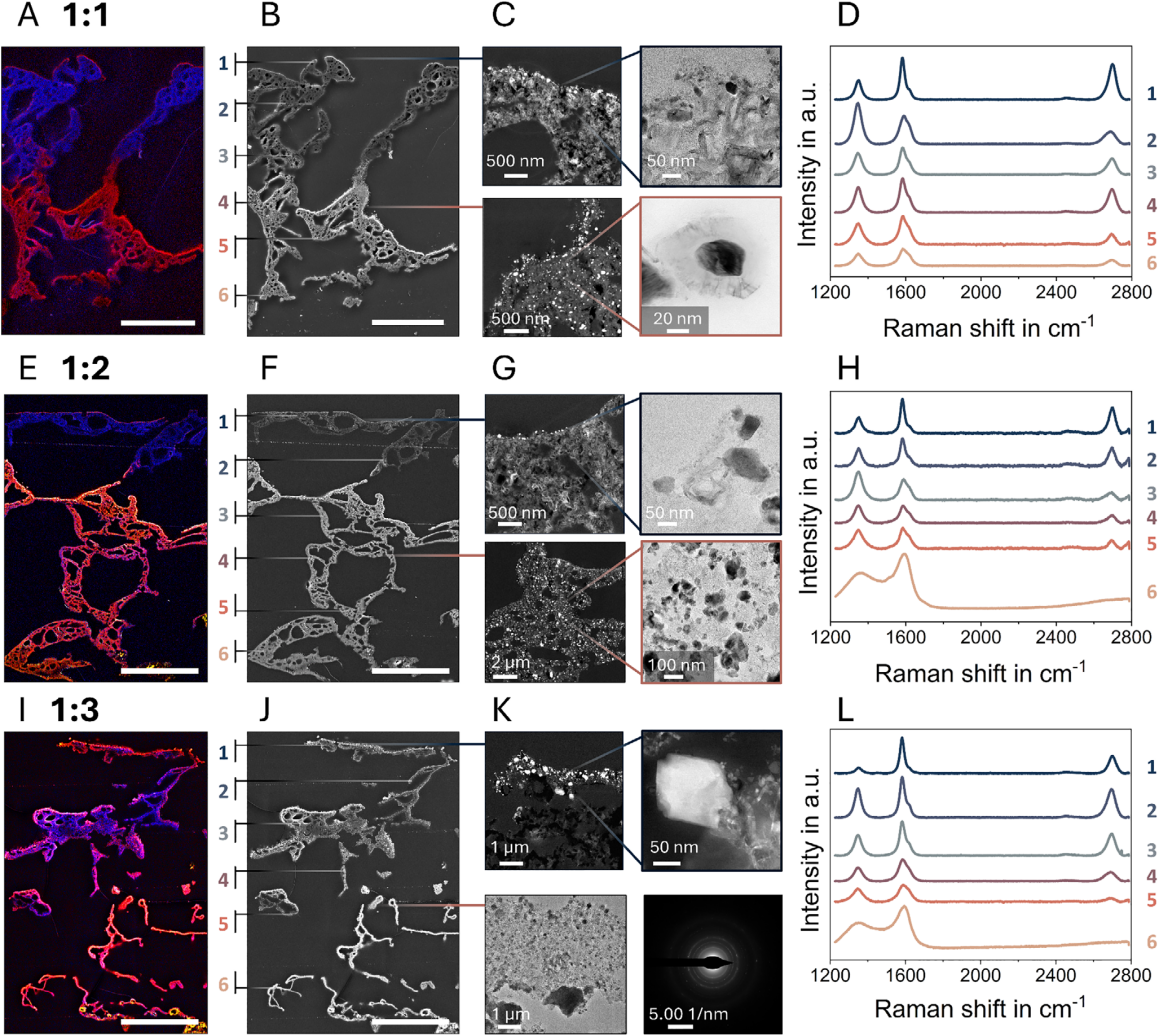
Cross‐sectional SEM images with corresponding EDS maps of the highly graphitized areas, showing details of the top and middle layers for three different TA:Fe ratios: A,B) 1:1, E,F) 1:2, and I,J) 1:3. Colors in the EDS maps: blue corresponds to carbon, red to iron, and yellow to oxygen. Scale bar: 25 µm. D,H,L) The corresponding Raman point measurements highlight the degree of graphitization across the cross‐section observed in all TA:Fe ratios. (Scanning) transmission electron microscopy ((S)TEM) images (ultra‐thin sections) show representative structural features of the top and middle layers for C) 1:1, G) 1:2, and K) 1:3. Agglomerations of magnetite (nano)particles are observed on the top surface of the highly graphitized layer for all TA:Fe ratios. The underlying layer is characterized by well‐dispersed, iron‐rich nanoparticles embedded in a graphitic matrix (C, G, K). The bright‐field image (C) shows a representative iron‐rich nanoparticle encapsulated in a graphitic shell, while the selected area electron diffraction (SAED) pattern in (K) confirms that the nanoparticles in the middle/bottom layer are magnetite.

To gain further insight into the nanoscale characteristics of the IC‐LIG electrodes, samples were embedded in a low‐viscosity resin, and thin cuts were prepared using an ultramicrotome for subsequent investigation via scanning electron microscopy (SEM) and (scanning) transmission electron microscopy ((S)TEM). SEM images with corresponding Energy Dispersive X‐ray Spectroscopy (EDS) maps (**Figure** **3**), showing the areal distribution of elemental carbon (blue), iron (red), and oxygen (yellow), highlight the peculiar structural arrangement resulting from the unidirectional laser treatment. Accordingly, all three IC‐LIG samples with different TA:Fe ratios exhibit a carbon‐rich top layer, while the underlying layers show an increasing iron signal. The structural features within the top layer are highly graphitized, exhibiting average interlayer spacings of ≈0.345, 0.343,^[^ ^45^ ^]^ and 0.348 nm for samples with TA:Fe ratios of 1:1, 1:2, and 1:3, respectively (Figure S6, Supporting Information). Highly graphitized nanoribbons were observed for all three different TA:Fe ratios, suggesting efficient iron‐catalyzed graphitization for all samples.

**Figure 4:**
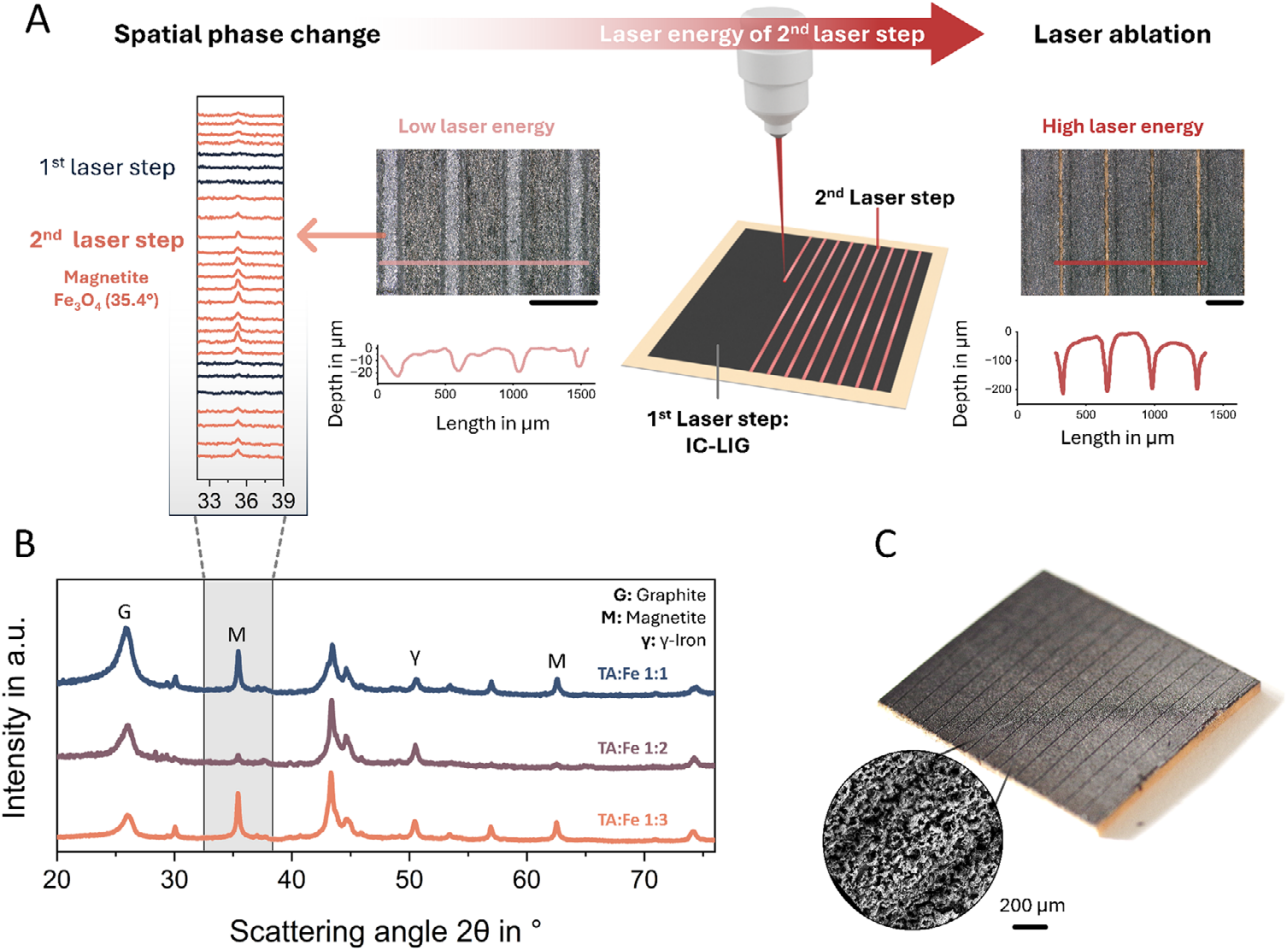
A) Schematic representation of the laser post‐treatment process, highlighting the application of a second laser step (double‐lased) on fully graphitized IC‐LIG electrodes (first laser step) to induce phase changes. This is evidenced by the wide‐angle X‐ray scattering (WAXS) line plot (A, left), where the emergence of the magnetite (Fe_3_O_4_) peak at 35.4° occurs without significant ablation, as demonstrated by the corresponding surface line profile. An ink composition with a TA:Fe ratio of 1:2 was selected, as it yields a high proportion of γ‐iron (metallic iron) evident from a distinct peak at 50.5° and the lowest amount of magnetite after initial laser‐induced graphitization, as shown in the B) Crystallographic analysis of scratched powder samples with TA:Fe ratios of 1:1, 1:2, and 1:3, together with reference peak positions. The diffractograms for all ink compositions are provided in Figure S8, Supporting Information. Increasing the laser energy during the second laser step leads to laser ablation (A), enabling the formation of sharp, deep grooves visible in the line profile (A, right). In (C), an electrode structured with high‐energy laser post‐treatment exhibits deep grooves that result in anisotropic conductivity, while the inset SEM image highlights the corresponding microscale structural changes.

The nanoparticles decorating the top layer were identified as magnetite particles (Figures 3C,G,K; Figure S7, Supporting Information). Notably, large magnetite particles agglomerate exclusively on the surface rather than within the top layer, a structural characteristic consistently observed across all investigated ink compositions. In contrast, the nanoparticles in the underlying layers are evenly distributed and embedded in a highly graphitized carbon matrix. This distribution appears to correlate with heat dissipation, driven by the unidirectional energy input of the laser, with particle sizes decreasing progressively toward the substrate. For all three TA:Fe ratios, we observed oxygen‐free, iron‐rich particles encapsulated in a multilayer graphitic shell (Figure 3C, Figure S6, Supporting Information). In accordance with the findings of our previous study^[^ ^45^ ^]^ and supported by X‐ray diffraction results (**Figures** **4** and S8, Supporting Information), these observations suggest that the iron particles are predominantly γ‐iron. X‐ray diffractograms (Figure S8, Supporting Information), together with Rietveld analysis (Figure S9, Supporting Information), further indicate that the γ‐iron phase content is highest in the 1:2 sample, whereas iron oxide (magnetite) and iron carbide are the prevailing phases in the 1:1 and 1:3 samples. Moreover, we observed a gradual decrease in graphitic carbon content with an increasing amount of iron in the precursor ink (Figure S9, Supporting Information). However, since the signals of certain iron phases such as iron carbide and γ‐iron can overlap, the values derived from the Rietveld analysis should be considered indicative of trends rather than absolute quantifications.^[^ ^32^ ^]^

### 2.3 Spatially Tunable Iron/Iron Oxide Phase through Laser Post‐Treatment

The amount of iron present in the precursor ink directly influences the overall composition of the IC‐LIG electrodes. Moreover, SEM and (S)TEM analysis revealed that unidirectional laser treatment further impacts the material, resulting in a mixture of different iron phases and nanoparticle sizes that follow the temperature zones across the electrode thickness.^[^ ^45^ ^]^ It can be reasonably assumed that the resulting iron phase depends on the laser energy applied. To explore this further, we investigated the potential for spatially tuning the iron phase through laser post‐treatment. Specifically, we applied a second, localized laser treatment by engraving single laser lines into fully graphitized IC‐LIG electrodes, as shown in **Figure** **4**A.

Following this approach, we demonstrate the spatial tunability of the iron/iron oxide phase within an IC‐LIG electrode (Figure 4A). To this end, we selected an ink composition of TA:Fe 1:2, as it yields a high proportion of γ‐iron (metallic iron) and the lowest amount of magnetite after initial laser‐induced graphitization (Figure 4B, Figures S8 and S9, Supporting Information). Figure 4A (left) shows the wide‐angle X‐ray scattering (WWAXS) line plots for single‐lased (dark blue; indicted as first laser step) and double‐lased (coral; indicated as second laser step) areas. Here, the characteristic magnetite peak at 35.4° is absent in single‐lased regions but clearly appears in double‐lased areas, indicating the oxidation of γ‐iron to magnetite upon repeated lasing.

It is noteworthy that such phase transition can be induced using a low‐energy laser post‐treatment, achieved through low laser power and a high defocus setting (5 mm). This configuration promotes oxidation without significant ablation, as confirmed by the surface line profile in Figure 4A. Despite this transformation, electrical resistance measurements revealed only minor changes, and the Raman map (Figure S10, Supporting Information) indicates minimal structural alterations. In contrast, high‐energy laser post‐treatment causes spatial ablation of the IC‐LIG electrode due to focused, high laser power, resulting in micro‐grooves on the electrode surface. These structural changes are highlighted in Figure 4C and its inset, which presents a microscale view via SEM imaging.

### 2.4 Influence of TA:Fe Ratios on the Electrical Properties

**Figure 5:**
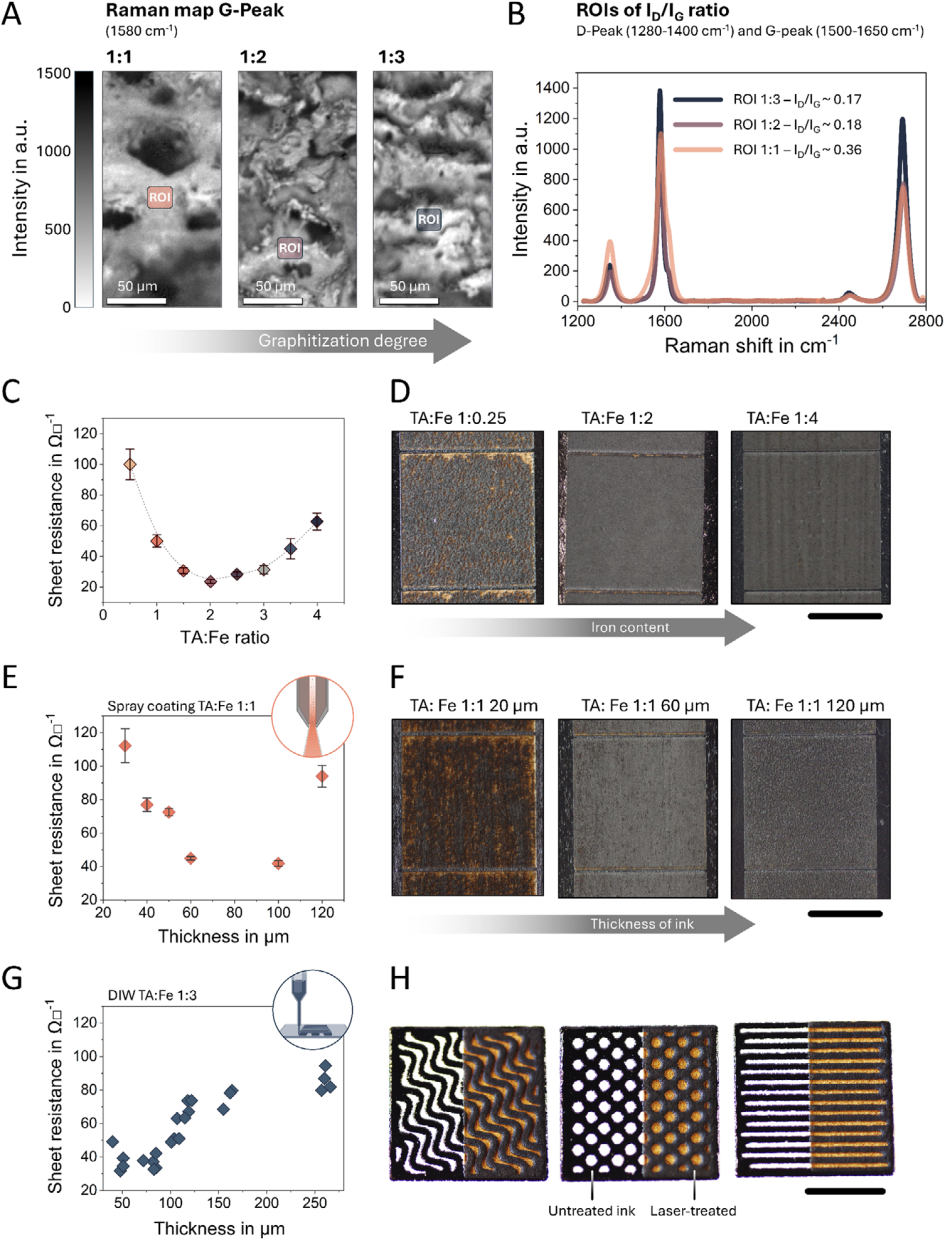
Top‐view Raman surface map A) of the G‐peak (1580 cm^−^), showing the exact positions (colored insets) of the spectra used for the corresponding regions of interest (ROIs) for each individual TA:Fe ratio. Averaged spectra for the ROIs are shown in (B), where the I_D_/I_G_ ratio was determined to highlight the degree of graphitization and the structural changes within the top surface layer as a function of the TA:Fe ratio. The dependence of ink composition on sheet resistance is shown in (C), with the 1:2 sample exhibiting the lowest value of 23.59 ± 1.2 Ω □^−^, accompanied by top‐view images highlighting the structural changes corresponding to the D) TA:Fe ratio. E) shows the dependency between spray coating thickness and the resulting sheet resistance, while top‐view images in (F) highlight the structural changes corresponding to the ink layer thickness. The relationship between the thickness of DIW‐printed ink layers and sheet resistance is shown in (G), and top‐view images in (H) illustrate the ability to print complex ink patterns (untreated ink, left) that can be subsequently laser‐engraved (laser‐treated, right). Scale bar: 10 mm.

The mutual relationship between the amount of iron in the precursor ink and the laser energy applied determines not only the resulting structure and phase composition of the IC‐LIG electrodes but also their electrical properties. This correlation is further supported by the Raman maps of the G peak (**Figure** **5**A), which provide insight into the top‐layer structure and highlight differences in the degree of graphitization among the three samples. Here, sample 1:1 is less graphitized, as shown by the representative ROI (region of interest) spectrum, which has an average I_D_/I_G_ ratio of ≈0.36 (Figure 5B). In contrast, as the iron content increases, the I_D_/I_G_ ratio decreases to about 0.18 for sample 1:2 and 0.17 for sample 1:3. At the same time, the G peak is narrower, while a reduction in the number of defects in the graphitic domains can be inferred since a decrease in the intensity of the D and D' peaks (shoulder of the G peak) is observed.^[^ ^55^ ^]^ However, the top layers of all three IC‐LIG samples show a high degree of graphitization in comparison with literature values on graphitic carbons, where the I_D_/I_G_ ratio is between 0.1 and 0.8.^[^ ^56^ ^]^ These values compare favorably with literature reports on graphitic carbons and contribute to the overall low sheet resistance observed. This relationship is further illustrated in Figure 5C, which shows a direct correlation between the TA:Fe ratio and sheet resistance. Among the tested ratios, the 1:2 TA:Fe sample demonstrates the lowest sheet resistance, ≈23.59 ± 1.2 Ω □^−^ on WPB, compared to both lower and higher ratios, provided that the laser parameters were the same for all samples. Additionally, the TA:Fe ratio was found to strongly influence IC‐LIG quality: low ratios (e.g., 1:0.25) led to irregular, highly resistive structures (>3000 Ω □^−^), while homogeneous and conductive IC‐LIG films were obtained with ratios between 1:0.5 and 1:4 (Figure 5C). However, it is important to note that the laser parameters were optimized for the 1:2 ratio; therefore, the electrical performance as well as the homogeneity of the IC‐LIG electrode surface structure was most uniform and coherent.

Achieving high conductivity in carbonaceous electrodes requires the formation of a stable percolation path through interconnected, highly graphitized domains. Surface Raman measurements (Figure 5A) indicate a high degree of graphitization for all samples, supported by (S)TEM analysis revealing the presence of graphitic nanoribbons (Figure 3C,G). However, cross‐sectional Raman measurements combined with EDS analysis (Figure 3) show that the high graphitization in the 1:1 sample is confined to the surface, whereas for the 1:2 and 1:3 ratio samples, a significant degree of graphitization extends beyond a thickness of 20 µm within the top layer, after which the Raman signal gradually shifts toward a spectrum characteristic of amorphous carbon for all samples. The probability of forming a stable percolation path is directly related to the depth of graphitization within the porous structure, which may explain the observed variation in conductivity between samples. The sample with a ratio of 1:1 has the highest overall porosity, yet its surface‐restricted graphitization hinders the efficiency of establishing a continuous conductive pathway. Conversely, the reduced conductivity observed in the sample with a ratio of 1:3 may be due to the combination of both, high porosity and the presence of larger magnetite agglomerates (Figure S7, Supporting Information), which further could interrupt the percolation path and thereby impede electrical conductivity.

To further understand the factors influencing IC‐LIG formation, the influence of ink layer thickness on IC‐LIG formation was systematically investigated across different application techniques, including spray coating and DIW. As shown in Figure 5C–G, the achievable thicknesses vary depending on the applied process parameters, such as printing speed, nozzle‐substrate gap, and applied pressure in the case of DIW, or nozzle distance and pressure for spray coating. However, homogeneous and well‐defined films were successfully deposited using all application methods investigated. Among them, spray coating demonstrated superior uniformity and scalability, particularly for large‐area coverage. This is evidenced by the low standard deviations observed across a range of target film thicknesses, indicating high reproducibility and process control. Specifically, the intended thicknesses and corresponding measured values were: 20 (21 ± 1), 30 (29 ± 2), 40 (40.7 ± 0.6), 50 (51 ± 2), 60 (60.7 ± 2.9), 100 (103.3 ± 2.1), and 120 µm (121 ± 2.7).

Thin coatings (<30 µm), particularly those obtained via spray coating or single‐layer screen printing (with a typical single layer thickness of ≈12 µm under the applied printing conditions), resulted in significant ablation and high sheet resistance upon laser‐treatment due to insufficient heat absorption capacity and partial substrate degradation (Figure 5E). For spray coating, the minimum viable layer thickness was found to be ≈30 µm, while screen printing with two consecutive layers reaching ≈25 µm yielded a sheet resistance of ≈300 Ω □^−^. Conversely, very thick layers (>100 µm) impaired heat dissipation during laser treatment, resulting in reduced electrical performance. This effect was particularly evident in DIW‐printed electrodes, which plateaued at sheet resistance values of ≈60–100 Ω □^−^ for layer thicknesses ranging from 110 to 260 µm, respectively. In comparison, spray‐coated samples exhibited a sheet resistance of 90 ± 6.5 Ω □^−^ at a thickness of 120 µm. Optimal LIG formation was consistently observed at intermediate ink layer thicknesses (≈80–100 µm) across all application methods.

These findings underscore the importance of carefully tuning both ink composition and film thickness to achieve optimal IC‐LIG performance. Notably, DIW not only enabled precise control over layer thickness but also facilitated the fabrication of complex, thick patterns that were successfully laser‐treated without any signs of ablation (Figure S4C, Supporting Information). The ink remained stable upon drying even at high thicknesses and on substrates such as plastic and WPB thanks to the inclusion of glycerol as a softener. As a result, the laser‐treated structures precisely followed the DIW‐printed patterns (Figure 5H).

As illustrated in the laser parameter studies (Figure S5, Supporting Information) conducted with a TA:Fe ratio of 1:2, the electrical properties can be readily tuned across a broad range by simply varying the power and speed during the laser treatment. This enables the IC‐LIG electrodes to be employed in a variety of applications, including as an electrical resistor and conductor. However, the utilization of an organic substrate imposes constraints on the energy density that can be applied through laser treatment. For example, the application of high‐power levels in conjunction with low engraving speeds can result in the formation of an irregular IC‐LIG electrode structure, which may lead to thermal damage and decomposition of the substrate (Figure S12, Supporting Information). Furthermore, humidity and temperature variations during operation can be critical. When exposed to higher humidity levels, cellulosic materials such as WPB undergo swelling, resulting in dimensional changes. Consequently, this could alter the interconnections between graphitic domains, influencing the electrical percolation path and, consequently the resistivity. However, in the absence of swelling‐induced substrate deformation, the IC‐LIG electrode exhibits minimal dependence of its electrical conductivity on humidity, with only slight changes in the reactance observed at elevated humidity (>80% relative humidity) for ink compositions with TA:Fe ratios of 1:1, 1:2, and 1:3, illustrated in Figures S13 and S14A, Supporting Information. As with humidity, there is a minimal impact of resistance due to external magnetic fields, with only slight alterations in reactance (Figure S15, Supporting Information). However, it is noteworthy that the response to an external magnet is comparable to the results reported in a recent study,^[^ ^57^ ^]^ as shown in Figure S16, Supporting Information, in which the powder sample scratched from an IC‐LIG electrode is responsive to a permanent magnet.

With regard to temperature, semiconductor‐like behavior is observed, with a decrease in resistivity and reactance at increasing temperatures (Figure S14B, Supporting Information). This could be attributed to an increase in the mobility of free electrons in the conduction band with rising temperature, analogous to that observed in graphitic carbon samples.^[^ ^58^, ^59^, ^60^, ^61^, ^62^, ^63^ ^]^ Moreover, the temperature dependence of resistance can be expressed by a complex temperature coefficient, indicating both resistive and reactive components. Here, the extracted temperature‐dependent resistance equations for the three different IC‐LIG electrodes follow the corresponding equations:

- TA:Fe 1:1 R(T[K]) = (−1.09+ j × −2.01) mΩ K^−1^ × T
- TA:Fe 1:2 R(T[K]) = (−0.977+ j × −2.09) mΩ K^−1^ × T
- TA:Fe 1:3 R(T[K]) = (−1.05+ j × −2.16) mΩ K^−1^ × T

The negative real components, such as −1.09, −0.977, and −1.05 mΩ K^−1^, indicate a decrease in resistance with increasing temperature, characteristic of the semiconducting behavior of graphitic carbon materials.^[^ ^58^, ^59^, ^60^, ^61^, ^62^, ^63^ ^]^ Meanwhile, the imaginary components also suggest a temperature‐dependent reactance, possibly influenced by the iron content. A comparison of the investigated TA:Fe ratios (1:1, 1:2, and 1:3) reveals that higher iron content results in a more pronounced reactance change, further emphasizing its use for prospective sensor applications. Notwithstanding the aforementioned influences, the electrodes demonstrate notable robustness, particularly considering the hygroscopic substrate (WPB) used.

### 2.5 Current Collector‐Free Electrode Design and Electrochemical Characterization

**Figure 6:**
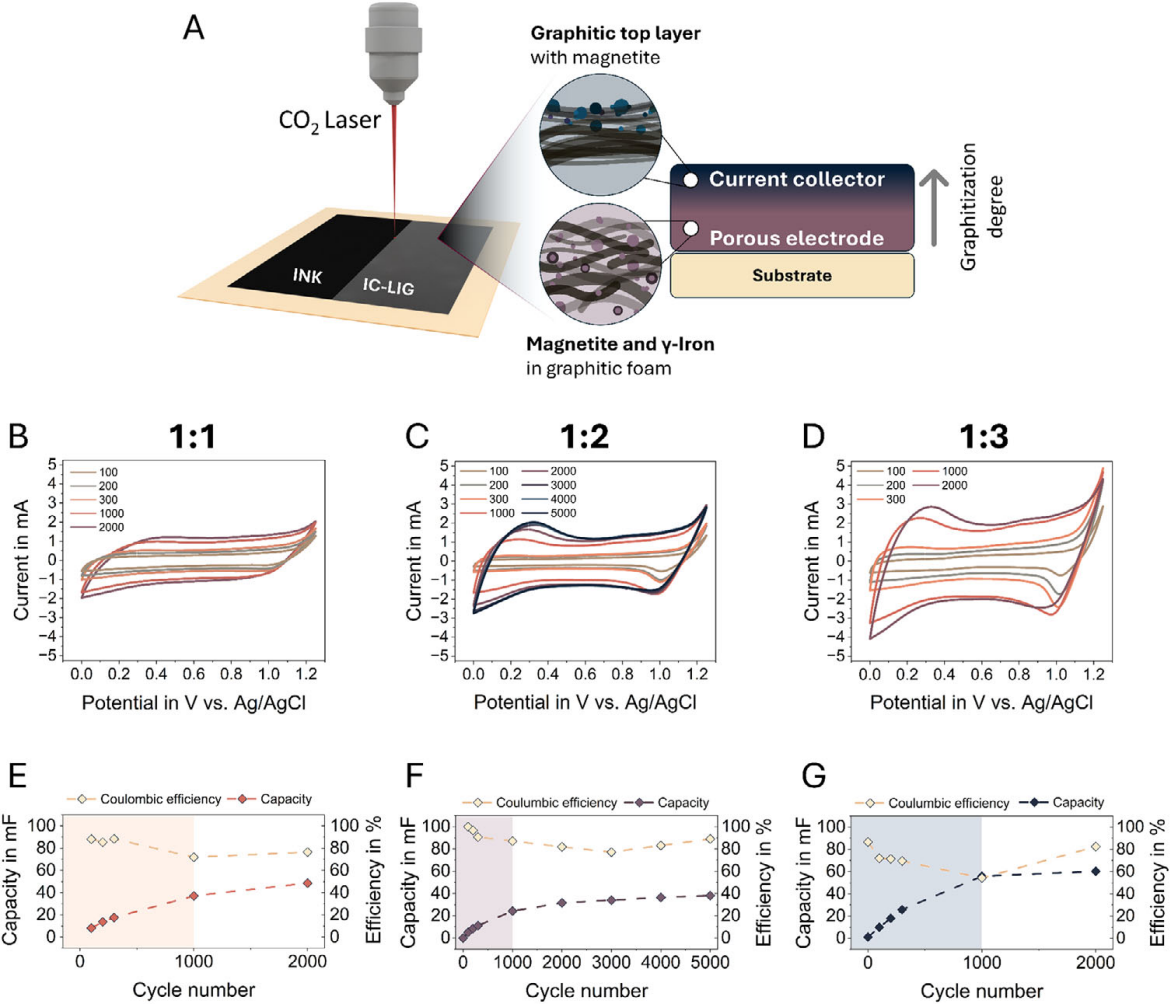
A) Schematic representation of the novel multilayer electrode design comprises the highly graphitized top layer as current collector (blue layer), with the subsequent layers representing the active hybrid electrode (purple layer) composed of an iron‐carbon composite. The electrochemical analysis of IC‐LIG electrodes with TA:Fe ratios of B–G) 1:1, 1:2, and 1:3. Capacitive behavior, which is similar to that of an EDLC, is reflected in the CV curves for the 1:1 sample (B). In contrast, the 1:2 and 1:3 samples display pseudo‐capacitive behavior as a result of faradaic reactions (C,D). All CV curves (50 mVs^−1^) demonstrate electrochemical activation, resulting in an increase in capacitance and current window over the course of the electrochemical cycling, evidenced by the calculated areal capacitance and resulting coulombic efficiency over cycling (E–G).

Given the direct correlation between graphitization and electrical conductivity, it is reasonable to assume that the top layer, being highly graphitized, is primarily responsible for electrical conductivity. Nevertheless, the underlying layers, which feature an amorphous carbon structure as indicated by Raman measurements (Figure 3D,H,L), also contribute to the overall electrical conductivity. Structural analysis of the cross‐section, including EDS and Raman measurements (Figure 3), reveals a graphitization gradient at the interface, suggesting a continuous electrical connection between the layers. We identified this as an opportunity to introduce a functional separation between the layers, which could open new avenues for electrochemical applications, particularly in energy storage. This approach enables the development of a novel multilayer electrode design that eliminates the need for traditional current collectors, typically made of metal^[^ ^18^ ^]^ or graphene.^[^ ^11^, ^13^ ^]^ Instead, the highly graphitized top layer serves as an effective current collector (**Figure** **6**A, blue layer), while the underlying layers function as the active hybrid electrode (Figure 6A, purple layer), composed of an iron‐carbon composite. Thus, the IC‐LIG process eliminates the need for solvents, binders, and additives (carbon black and graphite powder) necessary for traditional electrode manufacturing,^[^ ^18^, ^39^ ^]^ significantly reducing both material consumption and energy‐intensive processing steps.

To investigate the functionality of such a multilayer electrode design, electrochemical measurements were conducted in a three‐electrode cell using 1 M NaCl aqueous solution as the electrolyte. The electrodes were produced using 1:1, 1:2, and 1:3 ratio inks on electrochemically inert PET foil (≈750 µm thickness) instead of WBP, to avoid the risk of electrode delamination due to swelling of the latter in the aqueous electrolyte. The stability of the IC‐LIG electrodes was further assessed using a qualitative peel test with Scotch tape, which showed minimal ablation, as shown in Figure S4, Supporting Information and supported by Movie S2, Supporting Information. As a result, the sheet resistance only marginally increased from 31.2 ± 0.67 Ω □^−^ to 33.38 ± 0.1 Ω □^−^ (*n* =  3; TA:Fe 1:3), indicating strong mechanical integrity of the laser‐induced graphene layer.

Figure 6B–D highlights the outcome of the initial 2000 cycles (for sample 1:2 5000 cycles) of the cyclic voltammetry (CV) analysis at a constant scan rate of 50 mVs^−1^ over an operating window of 0 to 1.25 V. Depending on the TA:Fe ratio, two different charge storage mechanisms were observed. The sample with the lowest iron content, with a TA:Fe ratio of 1:1, exhibits EDLC. However, as CV analysis progresses, there is a significant increase in the measured current window from ≈−0.5 to 0.5 mA (after 100 cycles) to −1.4 to 1.4 mA (after 2000 cycles), which is reflected in an increase of the areal capacitance from 2.05 to 12.15 mF cm^−^^2^ at 0.025 mF cm^−^^2^ (Figure 6B–E). In contrast, the initial phases of the CVs of the samples with a ratio of 1:2 and 1:3 are characterized by comparable EDLC behavior; however, between 300 and 1000 cycles, it changes to a redox reaction‐based pseudocapacitive behavior (Figure 6C,D). Similarly, for the 1:1 ratio sample, a significant increase in the measured current window is observed, reflected in an increase in areal capacitance from 1.27 to 9.53 mF cm^−^^2^ for the sample with a ratio of 1:2 and from 2.5 to 15.06 mF cm^−^^2^ for the sample with a ratio of 1:3 at 0.025 mF cm^−^^2^ (Figure 6F,G). Interestingly, the capacity, as well as the coulombic efficiency, remain constant over the subsequent cycles (Figure 6E–G).

**Figure 7:**
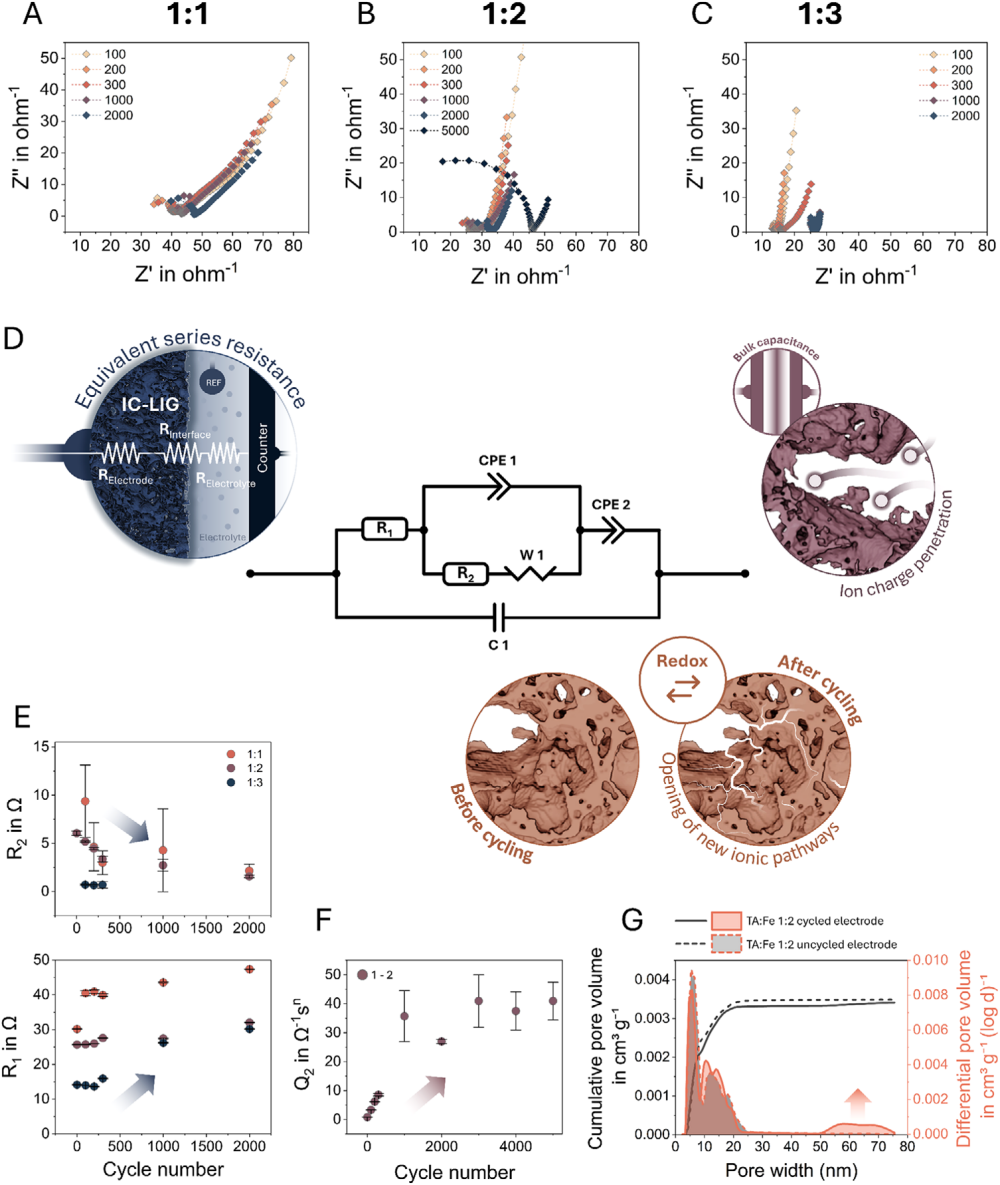
The electrochemical impedance spectroscopy (EIS) measurements of the samples with TA:Fe ratios of A–C) 1:1, 1:2, and 1:3 indicate an increase in the equivalent series resistance (ESR) as reflected by a curve shift toward higher real impedance values with an increase in the number of electrochemical cycles. The corresponding equivalent circuit model (ECM) in (D) illustrates the electrochemical and structural influences by changes to corresponding individual circuit elements. The increase in R_1_, which represents the resistance of the IC‐LIG electrode itself and contact resistance (R_1_ = R_electrode_ + R_electrolyte_), as well as the decrease in R_2_, which represents the charge transfer resistance between the electrode and the electrolyte (R_2_ = R_interface_), are a consequence of E) structural alterations and Faradaic reactions. Q_2_ (CPE_2_) represents F) the bulk capacitance. The increase of Q_2_ is directly related to the opening of new ionic pathways, which increases the accessibility of the redox sides, and is further supported by BET (N_2_) analysis G) comparing uncycled and cycled electrodes. This analysis reveals a shift in pore size distribution (between 5 and 20 nm) and the significant emergence of pores in the 50–75 nm range upon electrochemical cycling.

As with the changes observed in the CV analysis, the galvanostatic charge‐discharge (GCD) curves show an increase in charge and discharge time, while the curve changes from a shape more similar to an ideal supercapacitor to a curve distortion possible due to faradaic reactions (Figure S17A, Supporting Information). Furthermore, within the Nyquist plot derived from electrochemical impedance spectra (EIS) (**Figure** **7**A–C), which were recorded between cycles of capacitive charging and discharging, a shift toward higher real impedance values with increasing number of cycles for all samples tested was observed. Notably, the extracted value of R₁ from the equivalent circuit model supports the increase in equivalent series resistance (ESR) observed in the GCD measurements, while the shift of the x‐axis intercept in the Nyquist plots qualitatively indicates higher overall resistance.

To gain further insight into electrochemical behavior, including the observed increase in capacity during CV and the change in ESR, an equivalent circuit model (ECM) was used to relate these characteristics to physical changes and potential redox reactions that could occur during electrochemical measurements. The ECM consists of a modified Randles circuit (Figure 7D), in which the ideal capacitor element typically used has been replaced by a constant phase element (CPE), more representative of porous and rough electrodes. An additional CPE was added in series with the Randles circuit to account for bulk processes, as ions penetrate the porous structure of the electrode, as illustrated in Figure 7D, while a capacitor was added in parallel with the entire circuit to account for the cell capacitance (C_1_), to take into account the capacitance of the liquid medium as well as parasitic capacitances.

Similar to the changes in the GCD and the shift in the Nyquist plot, we observed an increase in resistance R_1_, which represents the resistance of the IC‐LIG electrode itself as well as the contact resistance and electrolyte resistance (R_1_ = R_electrode_ + R_electrolyte_; Figure 7D). Notably, while we cannot directly separate R_electrode_ and R_electrolyte_ from the measurements, the observed shift toward higher resistance values strongly suggests that R_electrode_ plays a comparable or even dominant role in R₁. Given that the electrolyte composition and measurement setup remained constant across all experiments, significant variations in R_electrolyte_ are unlikely.

At the same time, the resistance R_2_, which represents the charge transfer resistance, for example, between the interface of the electrode and the electrolyte, decreased over time, indicating a pronounced and increasing electrochemical activity. The relatively low resistance of R_2_ is an indication of electrochemical activity, since electrodes without redox activity are characterized by a charge transfer resistance in the order of kOhm to MOhm. Moreover, it is noteworthy that a semicircular shape in the Nyquist plot was observed for the 1:2 ratio sample after 5000 cycles. A comparable, yet inverse behavior was recently documented in the case of a manganese oxide (MnOx) pseudocapacitor electrode, wherein enhanced interfacial conductivity between the current collector and the MnOx electrode resulted in a shift toward lower real impedance values, thus lower ESR.^[^ ^64^ ^]^ Since the ESR is directly related to the electrical percolation path of the IC‐LIG electrode, we consider structural changes resulting from electrochemical cycling to be of consequence. This is further supported by an increase in sheet resistance from ≈20 to 35 Ω□^−1^ measured before and after electrochemical analysis. Additional BET (N_2_) analysis of cycled electrodes (Figure 7G), directly compared to uncycled counterparts, further supports this interpretation. The data reveal a shift in pore size distribution within the 5–20 nm range and a pronounced emergence of pores between 50 and 75 nm, suggesting enhanced ion accessibility and the formation of new diffusion pathways.

Figure S17D, Supporting Information compares CPE_1_ with CPE_2_ for the three samples after the first 100 cycles by their Q values, a complex number that can be interpreted as indicative of increased or decreased resistive and capacitive properties, contingent on its variable N. The variable N can take values between 0 and 1, while for *N* = 0, the CPE is purely resistive (*Q* = 1/(R)), whereas for *N* = 1, the CPE is purely capacitive, and the Q value is equal to the real capacitance. Given that the values of N were at least 0.7 and, in fact, above 0.9 for the majority of data points, it is reasonable to assume that the Q values for both CPEs represent their capacitance. Here, the Q_1_ values (CPE 1) show an order of magnitude lower capacitance, within the nF range, compared to Q_2_. This is well in line with the proposed model where Q_1_ represents the surface capacitance while Q_2_ represents the bulk capacitance. Here, the values of Q_2_ are considered to be relatively high because of the porous structure of the IC‐LIG electrode, while they can be considered to represent the total capacitance of the samples. The changes in bulk capacitance throughout electrochemical cycling are further illustrated by a representative plot of a sample with a TA:Fe ratio of 1:2 over 2000 cycles. After an apparent electrochemical activation, represented by a steep increase in capacitance within the first 1000 cycles, the values plateau at ≈40 mF (Figure 7F).

In accordance with the structural investigations, the ECM further supports the interpretation that structural rearrangements occur during electrochemical cycling. It is therefore reasonable to conclude that the opening of new ionic pathways during electrochemical cycling is a major mechanism responsible for the observed increase in capacitance.^[^ ^65^, ^66^, ^67^ ^]^ This is reflected in the increased resistance of R₁, which suggests the partial disruption of electronic pathways. In addition, the increased redox activity observed in the decrease of R_2_ may be related to the availability of more redox active sites. Furthermore, Figure S17F, Supporting Information shows an increase in the Warburg element (W₁) values for all samples, indicating greater diffusion limitations. This is likely due to ion penetration and intercalation associated with channel opening and increased tortuosity before and after cycling, as illustrated in Figure 7D.

A direct comparison of the capacity values between the samples demonstrates the influence of both the electrode composition and the changes in iron/iron oxide content, as well as their structure. This is particularly evident in the case of samples with a ratio of 1:1, which exhibit a high capacity but incorporate the lowest amount of iron/iron oxide, thus the lowest potential for redox reactions. Nevertheless, an examination of the specific surface area by means of dynamic vapor sorption (DVS) and N_2_ sorption measurements (Figure S18, Supporting Information) indicated that this sample exhibited the highest value of ≈187 m^2^g^−1^ (N_2_), which directly correlates with its capacity.

To contextualize the performance of the IC‐LIG electrodes developed in this study, Table S2, Supporting Information presents a comparative overview of key performance indicators (KPIs), including ESR, sheet resistance, areal capacitance, and surface area, benchmarked against literature on laser‐induced graphitization. Compared to LIG‐based microsupercapacitors without active materials, such as wax‐modified, paper‐derived LIG, which already achieves a notable capacitance of 5.76 mF cm^−2^ at 0.01 mA cm^−2^ in a PVA/H_2_SO_4_ gel,^[^ ^22^ ^]^ the IC‐LIG electrodes exhibit enhanced performance. The results demonstrate that the combination of high surface area and redox‐active iron species leads to a significantly improved capacitance of 15.06 mF cm^−2^ for the sample with a 1:3 ratio, even when using a simple 1 M NaCl electrolyte at 0.025 mA cm^−2^.

It is also worth noting that MoS_2_‐containing PI‐LIG devices using a 1 M NaCl/PVA gel reach 14 mF cm^−2^ at 0.1 mA cm^−2^,^[^ ^68^ ^]^ and FeCl_3_ crystal‐coated PI‐LIG microsupercapacitors achieve 719 mF cm^−2^ in PVA/H_2_SO_4_ at 1 mV s^−^.^[^ ^69^ ^]^ These comparisons emphasize the potential of electrolyte optimization to further enhance IC‐LIG performance and to explore redox activity beyond NaCl‐based systems. Overall, the IC‐LIG electrodes presented here offer a promising balance of performance, processability, and sustainability.

## 3 Conclusion

By producing current collector‐free hybrid carbon‐iron (oxide) electrodes using the IC‐LIG process, we have introduced a transformative approach to sustainable electrode fabrication. Through tuning the amount of iron(III) ions present in the bio‐based tannic acid–iron precursor ink, precise control over the ink's rheological properties is achieved. Leveraging this adaptability in the manufacturing process enables versatile application techniques, including spray coating, screen printing, and DIW, while ensuring compatibility with diverse substrates such as plastic films, wood veneers, and cellulosic materials. We systematically investigated the feasibility of employing these application methods to deposit inks with a wide range of thicknesses. Subsequent laser‐treatment of these coatings revealed a minimum thickness of ≈30 µm under the current laser parameters, while sheet resistance of 23.59 ± 1.2 Ω □^−^ on renewable wood‐based substrate was achieved. Among the application methods, Direct Ink Writing DIW stood out by enabling the fabrication of highly complex patterns. Notably, the resulting IC‐LIG electrodes exhibited excellent reproducibility and outstanding abrasion resistance, particularly on plastic substrates, highlight their robustness and suitability for practical applications. Crucially, iron plays a dual role throughout the process—from ink formulation and catalytic graphitization during lasing to functioning as an active redox component—reinforcing its central contribution to process efficiency. As a result, the IC‐LIG approach is streamlined during the development stage to meet industry requirements, such as large‐scale and flexible processing.

An in‐depth, multi‐scale structural analysis revealed that the subsequent unidirectional laser treatment results in a layered electrode arrangement for all TA:Fe ratios investigated. Specifically, the IC‐LIG electrodes consist of a highly graphitized top layer, while the underlying structure is a composite of iron oxide particles embedded in a porous graphitic foam. Interestingly, a simple low‐energy laser post‐treatment allows spatial tuning of the iron oxide phases, converting γ‐iron into magnetite, expanding the design possibilities. This combination of spatial control and compositional tuning supports the tailoring of electrochemical performance and opens up potential for catalytic applications such as hydrogen and oxygen evolution reactions.

This layered electrode configuration further enables a novel all‐in‐one multilayer electrode design, where the graphitized top layer serves as a current collector, and the iron‐carbon foam functions as a hybrid electrode. Compared to conventional electrode fabrication methods, this approach entails the production of a fully functional electrode directly on the substrate, bypassing energy‐intensive processing steps such as graphite powder production, slurry mixing, drying, and calendaring.^[^ ^18^, ^39^ ^]^ The high graphitization degree of the top layer, irrespective of the TA:Fe ratio, ensures efficient charge transport and eliminates the need for a metal current collector, thereby reducing material consumption, weight, costs, and enhancing the sustainability of electrode manufacturing. Adjusting the TA:Fe ratio further tailors the structure and electrochemical properties. For instance, a low TA:Fe ratio results in the highest surface area (≈187 m^2^g^−1^), directly correlated with high capacity. While low iron content favors EDLC behavior, higher iron content introduces pseudocapacitive characteristics via Faradaic reactions, allowing the charge storage mechanism to be tailored for diverse energy storage needs. In comparison to existing literature, the IC‐LIG electrodes developed in this study exhibit outstanding electrochemical stability and high areal capacitance, retaining 15.06 mF cm^−2^ after 5000 cycles at a TA:Fe ratio of 1:3, even in simple electrolytes such as 1 M NaCl, demonstrating their practical relevance (Table S2, Supporting Information). Further improvements in carbon yield and electrolyte compatibility, as well as electrolyte‐specific studies, will enable deeper understanding of the redox mechanisms and extend application potential. To gain more direct insight into the nature and evolution of redox‐active species, future work will focus on developing in situ and operando characterization methods (e.g., SAXS,^[^ ^70^ ^]^ Raman spectroscopy^[^ ^71^ ^]^). These approaches will allow us to monitor phase changes during electrochemical cycling and better correlate them with electrochemical performance. Future efforts should also focus on integrating renewable substrate materials (e.g., wood, cellulose) into full device fabrication workflows.

Altogether, the IC‐LIG approach represents a highly adaptable fabrication platform that combines sustainable materials, functional versatility, and manufacturing simplicity, thus offering a promising foundation for next‐generation energy storage, harvesting, sensing, and sustainable electronic devices.

## 4 Experimental Section

### Materials

Tannic acid (source: Chinese natural gall nuts), ammonium iron(III) citrate (technical grade), and glycerol (99 + %) were purchased from Sigma‐Aldrich. All chemicals were used as received. Deionized water (DI) was used unless otherwise stated. As substrate material, acid‐free wood pulp board (Gebr. Bühler AG Zürich, CH) produced from mechanical pulp of pine, spruce, and birch was used. The wood pulp board had a double‐sided smooth surface and a grammage of 750 g m^−2^, while its thickness was ≈1.5 mm. Commercial polyethylene terephthalate (PET) foil with a thickness of ≈750 µm (PLU 603, Gutta) was used for the electrodes, while screen prints were applied on 125 µm PET foil (Hostaphan GN). Prior to ink application for electrode manufacturing, the foils were thoroughly washed with soap.

### Preparation of the Precursor Inks

The basis of the recipe for the precursor iron‐tannic acid inks follows a recently published recipe^[^ ^41^ ^]^, with the only significant modification being the variation of the tannic acid to iron(III) ratio (TA:Fe). All other components remained constant. Here, TA:Fe refers to the stoichiometric (molar) ratio between tannic acid and Fe^3^⁺ ions, with the iron supplied by ammonium iron(III) citrate. The different ink compositions were calculated based on molar ratios, and the corresponding values are provided in Table S1, Supporting Information. To prepare the ink, tannic acid (33 g) was dissolved by its addition in small portion sizes to 77 g of water, with stirring at ≈500 rpm. Subsequently, 8 g of glycerol and the requisite amount of ammonium ferric citrate for the targeted TA:Fe ratio were added in small portions under continuous stirring to ensure complete dissolution. The resulting iron tannic acid ink was stored at room temperature until required.

### Application Method

The iron‐tannic acid ink was applied on WPB using a commercial paintbrush (GraduateXL, flat, Daler Rowney, UK). Three layers of ink were applied to obtain homogenous coating, resulting in a about 100 µm thick layer, measured with a micrometer (Mitutoyo). The applied ink amount was measured with a laboratory scale (AE 163, Mettler, CH) resulting in 170 gm^−2^. The samples were then left for drying for at least 12 h at 20 °C and 65% RH before use.

To demonstrate the rheological properties of inks with different TA:Fe ratios, three application methods were employed: spray coating, screen printing, and DIW. Notably, no rheology modifiers or other additives were used. For spray coating, a TA:Fe ratio of 1:1 was applied using a commercially available airbrush (Evolution, Harder & Steenbeck, DE) with 0.2 mm and 0.4 mm nozzles and compressed air at a working pressure of ≈2–2.5 bar. For screen printing, a TA:Fe ratio of 1:2 was used with a semi‐automatic EKRA screen printer. A 120–34 mesh screen (120 threads per cm, 34 µm thread diameter) was employed. Multiple layers were applied through consecutive prints, with short drying intervals (<5 min) between each step. The printing parameters included a print and flood speed of 170 mm s^−^, a pressure of 1.7 bar (83 N), and a 2 mm printing gap. For extrusion‐based 3D printing using direct ink writing (DIW), a TA:Fe ratio of 1:3 was used. Prior to printing, the ink was homogenized in a speed mixer (SpeedMixer DAC 150.1 FVZ) for 5 min at 2000 rpm. Small amounts of water were added as needed to fine‐tune the rheological properties, depending on environmental and printing conditions. Printing was performed at room temperature using a DIW system (Envisiontec 3D‐Bioplotter Manufacturer Series, Germany). A 0.41 mm conical nozzle (H. Sigrist + Partner AG, CH) was used. The Bioplotter operated with a gauge pressure of 2.5–3.5 kPa and a nozzle speed of 12–14 mm s^−^. Printed samples were dried at room temperature.

### Laser Treatment

The samples were treated with a commercially available 10.6 µm CO_2_ laser engraver (Speedy 400, Trotec), which has a maximum power of 80 W and a maximum scan rate of 4.3 m s^−1^. The parameters utilized for the analysis of the samples, both structural and electrochemical, included power values of 7.5% with a scan rate of ≈200 mm s^−^, an image density of 1000 pulses inch^−^, and a defocus of up to 5 mm (resulting beam diameter of about 450 µm). In the parameter study, the defocus and image density were maintained at the same levels; however, the power and speed values were adjusted accordingly (Figure S5, Supporting Information).

For the laser post‐treatment, low energy (LowE) treatment with power values of 3%, scan rate of ≈430 mm s^−^, and a defocus of up to 5 mm, while for high energy (HiE) treatment power values of 10%, scan rate of ≈43 mm s^−^, and no defocus were used.

### Characterization Techniques

The rheological behavior of the inks was characterized using an MCR 501 Anton Paar rheometer with two different measuring geometries. For inks with lower iron content (1:0.25, 1:0.5, 1:1, 1:1.5, 1:2, and 1:2.5), a cup‐bob geometry (CC27) with a 0.5 mm gap was used. Inks with higher iron content displayed paste‐like behavior and exhibited too high viscosity for the cup‐bob geometry. Therefore, for inks with higher iron content (1:3, 1:3.5, and 1:4), a 25 mm diameter plate‐plate setup with a 0.5 mm gap was chosen. A Peltier temperature device was used to maintain a constant temperature of 25 °C during tests and to minimize evaporation effects. Flow viscosity was measured by varying the rotational shear rate from 0.01 to 1000 s^−1^ with a logarithmic sweep at a fixed amplitude of 1%.

Microstructural measurements including the line plots for laser‐ablated samples were performed using a digital optical microscope (Keyence VHX‐6000, Keyence, JP) and open source image analysis software ImageJ (1.53e).

Analytical (scanning) transmission electron microscopy ((S)TEM) was performed on an FEI Talos F200X operated at 200 kV acceleration voltage. The specimens were dried at 103 °C for 12 h and embedded in Spurr low viscosity embedding resin (Sigma Aldrich). Ultrathin sections (≈100 nm) were prepared on an ultramicrotome (Ultracut E, Reichert‐Jung) equipped with a diamond knife (Diatom) and flattened using chloroform vapor. The ultrathin sections were deposited on copper grids with a ≈5 nm continuous carbon supporting film. Elemental content distribution mapping was carried out by energy‐dispersive X‐ray spectroscopy (EDS) STEM spectrum imaging with a windowless Super‐X EDS system. The EDS spectra were acquired up to 20 keV with a spectral resolution of 10 eV per channel and an acquisition time of 300 s to 1200 s. Selected‐area electron diffraction (SAED) patterns were acquired with a 40 µm SAD aperture corresponding to a diameter of ≈800 nm in the object plane. The corresponding reference cards for analysis of the SAED patterns are PDF 04‐015‐2407 for graphite and PDF 00‐019‐0629 for magnetite.

High‐resolution block‐faces images of carbon‐coated samples (2 nm) were taken with a scanning electron microscope (SEM, SU5000, Hitachi Ltd., Japan) driven by an accelerating voltage of 10 kV (SE mode), while the local concentrations of iron, and oxygen were mapped with the energy‐dispersive X‐ray detectors (Oxford Ultim Max 100 EDS detectors mounted at 90° to each other, Oxford Instruments, UK).

A PANalytical X'Pert PRO MPD diffractometer using monochromated Cu Kα1 radiation (40 kV, 45 mA) in Bragg–Brentano geometry was used. Samples for X‐ray diffraction (XRD) were prepared by scratching off the LIG from samples of the size 20 × 20 mm^2^, further ground using an agate pestle and mortar, and mounted on a low background Si sample holder. By optimizing the pulse height distribution limit (PHD, lower limit set to 60%, upper limit to 80%), the fluorescent background was suppressed. The diffractograms were collected from 5° to 80° with a total acquisition time of 5 h and a step size of 0.0334°. A background was fitted and subtracted, peaks were indexed, the crystallite size was calculated, and Rietveld refinement was performed using HighScore Plus. The corresponding reference cards from the PDF4 database^[^ ^52^ ^]^ are 00‐041‐1487 for graphite,^[^ ^53^ ^]^ 00‐058‐1638 for carbon nanotubes,^[^ ^54^ ^]^ 00‐006‐0696 for α‐iron, 04‐003‐1443 for γ‐iron, 00‐019‐0629 for magnetite, and 00‐35‐0772 for iron carbide.

Wide‐angle X‐ray scattering patterns were recorded using a Xenocs Xeuss 3.0 in house SAXS/WAXS diffractometer, equipped with a Cu Kα and a Dectris Eiger 1 M 2D X‐ray detector. The specimen was placed on the solid sample holder (Xenocs) and placed inside the sample chamber, where it was positioned at a sample detector distance of 50 mm (calibrated using a lanthanum hexaboride standard). The sample was scanned using a 0.25 × 0.25 mm^2^ beam and a step size of 0.1 mm with an exposure time of 2700 s per image (using the line eraser mode; two images are collected with 1350 s exposure time at slightly shifted detector positions and afterward merged). The 2D scattering data was then azimuthally integrated to obtain the 1D scattering pattern. All data handling was performed using Xenocs XSACT Software (Version 2.9).

For the nano‐CT acquisition, cubic samples with the size of 1 × 2 × 1.5mm^3^ were cut with a CO_2_ laser engraver (Speedy 400, Trotec) and mounted on a needle tip with 2K epoxy (Z‐Poxy, ZAP, USA). They have been scanned focusing through a zoom scan on the interface surface area using a EasyTom XL Ultra 230 160 micro/nano CT scanner (RX Solutions, Chavanod, FR). The scanner operated at 60 kV and 165 µA. The samples were scanned over a 360° rotation with a step size of 0.1°, frame average of 3 and exposure time of 2 s. Nominal resolution was set at 250 nm voxel size. CT images were reconstructed using X‐Act (RX Solutions, Chavanod FR) software. Reconstructed 2D CT slices were then analyzed using AVIZO software (Thermo Fisher Scientific, USA). This software was used both to create 3D models, the MIP projections and to calculate porosity. Specifically, for porosity calculations, the percentage of porosity was calculated by global threshold as the ratio of the pore volume of each layer to the total volume of the corresponding layer's rectangular bounding box.

Raman spectroscopy was performed with a confocal Raman microscope (Renishaw InVia) using a 532 nm laser, an objective (Zeiss, 60× water immersion) and a 1800 l mm^−1^ grating. The integral exposure time was 3 s for 50 accumulations covering a spectral range of ≈1220–2790 cm^−1^ with about 0.5 mW laser power (1% of 48 mW) for single‐point measurements. As mapping parameters, an integration time of 1 s (single spectrum acquisition) with 5 mW laser power (5% of 101 mW) and a step width of 1 µm was used in the Map image acquisition mode over an area of about 100 × 200 µm^2^. After data acquisition, a baseline correction and cosmic ray removal filter were applied using the Wire 3.7 software (Renishaw UK). For chemical imaging, data were exported into CytoSpec (v. 2.00.01), a commercially available MatLab‐based software.

Thermogravimetric analysis (Q50, TA instruments, New Castle, USA) with a platinum sample pan was used to compare the effects of an inert nitrogen atmosphere (N_2_) and an oxygen‐rich atmosphere (synthetic air; N_2_ + 20% ± 2% O_2_) during thermal treatment (up to 1000 °C, 10 °C min^−1^) of the untreated iron‐tannic acid ink.

The specific surface area (SSA) of the samples was determined through a multipoint Brunauer‐Emmett‐Teller (BET) analysis of nitrogen gas sorption isotherms (BET N_2_), which were performed at 77 K by using a high vacuum adsorption analyzer (autosorb iQ, Quantachrome, US). Prior to the measurements, the samples were degassed at 100 °C under vacuum for at least 24 h.

Dynamic water vapor adsorption and desorption were measured by an automated sorption balance device (DVS Advantage ET85, Surface Measurement Systems Ltd.). Scratched‐off IC‐LIG electrode powder was first dried for 10 h at 60 °C and at a partial water vapor pressure of p/p0 = 0. The samples were then exposed to ascending p/p0 steps of 0.05 starting from 0 to 0.95 with an additional step at 0.98 for adsorption and then descending in the same manner for desorption at 25 °C. Equilibrium in each step was defined either as mass change per time (dm/dt) of less than 0.0005% min^−1^ over a 10 min window or a maximal time of 1000 min per step. The samples were exposed to a flow rate of 200 sccm, while as carrier gas nitrogen (5.0 grade) was used.

Sheet resistivity measurements were conducted using a four‐point probe (Signatone 302, spring‐loaded (0.6 N) round‐headed gold contacts) equipped with a source measure unit (2450, Keithley Instruments, US). Sample sizes used for sheet resistance measurements were *n*  =  16 for brush‐coated samples, evaluated for each TA:Fe ratio ranging from 1:0.5 to 1:4 in 0.5 increments; *n*  =  6 for spray‐coated samples with coating thicknesses of 30, 40, 50, 60, 100, and 120 µm; and a total of 24 DIW‐fabricated samples with thicknesses ranging from 40 to 260 µm were analyzed. For the electrical resistance measurements, an LCR meter (Keysight E4980A) with a custom setup (Figure S13, Supporting Information) was connected via the USB‐GPIB interface to a computer running a Matlab (v. R2022b) script which performed an impedance measurement in the frequency range 20 Hz to 20 MHz. The influence of humidity was measured in a climatic chamber between 10 and 80% relative humidity, while the influence of a magnetic field was characterized using neodymium magnets (300 mT).

All electrochemical measurements including cyclic voltammetry, galvanostatic charge‐discharge cycles (chronopotentiometry), and electrochemical impedance spectroscopy were performed using a multichannel potentiostat (OctoStat, Ivium) in 1 M aqueous NaCl solution. IC‐LIG electrodes were used as the working electrodes (WE) together with a stainless‐steel mesh as counter electrode (CE) and an Ag/AgCl reference electrode (RE) within the three‐electrode electrochemical measurements setup equipped with an 3D printed holder for all electrodes. For calculating the gravimetric properties, sample mass was measured using a laboratory precision balance (AE 163, Mettler‐Toledo GmbH, CH). For the ECM the software IviumSoft was used to fit the data from the electrochemical impedance spectroscopy (EIS). EIS measurements were conducted within the frequency range of 1 Hz to 100 kHz, however, the highest frequencies exceeded the capabilities of the equipment, resulting in the acquisition of data that was characterized by a high degree of noise. Consequently, the last five data points were excluded from all subsequent data analysis. For each fitting, the values of the circuit elements were obtained, along with an error value expressed as a percentage. Errors exceeding 200% were not included in the results.

Environmental conditions during IC‐LIG electrode fabrication and characterization were maintained in a climatized laboratory, with relative humidity ranging from 30% to 70% and temperature controlled at 20 ± 5 °C, unless otherwise specified.

In general, data was evaluated and illustrated using the software OriginPro 2019 (version 9.6.0.172, OriginLab Corporation, US) and Adobe Illustrator (version 28.4.1, Adobe Inc., US). The Scientific color map Lipari^[^ ^55^ ^]^ was used to prevent visual distortion of the data and exclusion of readers with color‐vision deficiencies.^[^ ^56^ ^]^

Schematic representations (Figure 1) were adapted from “Round bottom flask (3/4 liquid); 3D bioprinter; 3D printing nozzle (multilayered),” by BioRender.com (2024). Retrieved from https://app.biorender.com/biorender‐templates

## Conflict of Interest

The authors declare no conflict of interest.

## Supporting information



Supporting Information

Supplemental Movie 1

Supplemental Movie 2

## Data Availability

The data that support the findings of this study are available in the supplementary material of this article.
