# Supplementary material for: Iron‐Catalyzed Laser‐Induced Graphitization Enabling Current Collector‐Free Electrodes With Spatially Tunable Iron/Iron Oxide Phases
Source: Adv Mater. 2025 Aug 1;37(41):e08812. doi: 10.1002/adma.202508812 (PMC12531747; doi:10.1002/adma.202508812)
Supplement: Supplementary file 1 — Supporting Information [file ADMA-37-e08812-s001.docx]

Supporting Information

**Iron-Catalyzed Laser-Induced Graphitization Enabling Current Collector-Free Electrodes with Spatially Tunable Iron/Iron Oxide Phases**

*Christopher H. Dreimol ^1,2*^, Jesper Edberg ^3^, Ronny Kürsteiner ^1^, Maximilian Ritter ^1,2^, Sophie Koch ^1,2^, Annapaola Parrilli ^4^, Robert O. Kindler^1,2^, Robert Brooke ^3^, Susanna Tinello^5^, Sandro Stucki ^1,2^, Simon Bryner ^6^, Gerd Simons^6^, Guido Panzarasa ^1*^, Ingo Burgert ^1,2*^*

**Table S1** Overview of the used mass of ammonium iron (III) citrate for the different ink compositions with the corresponding TA:Fe ratio.**
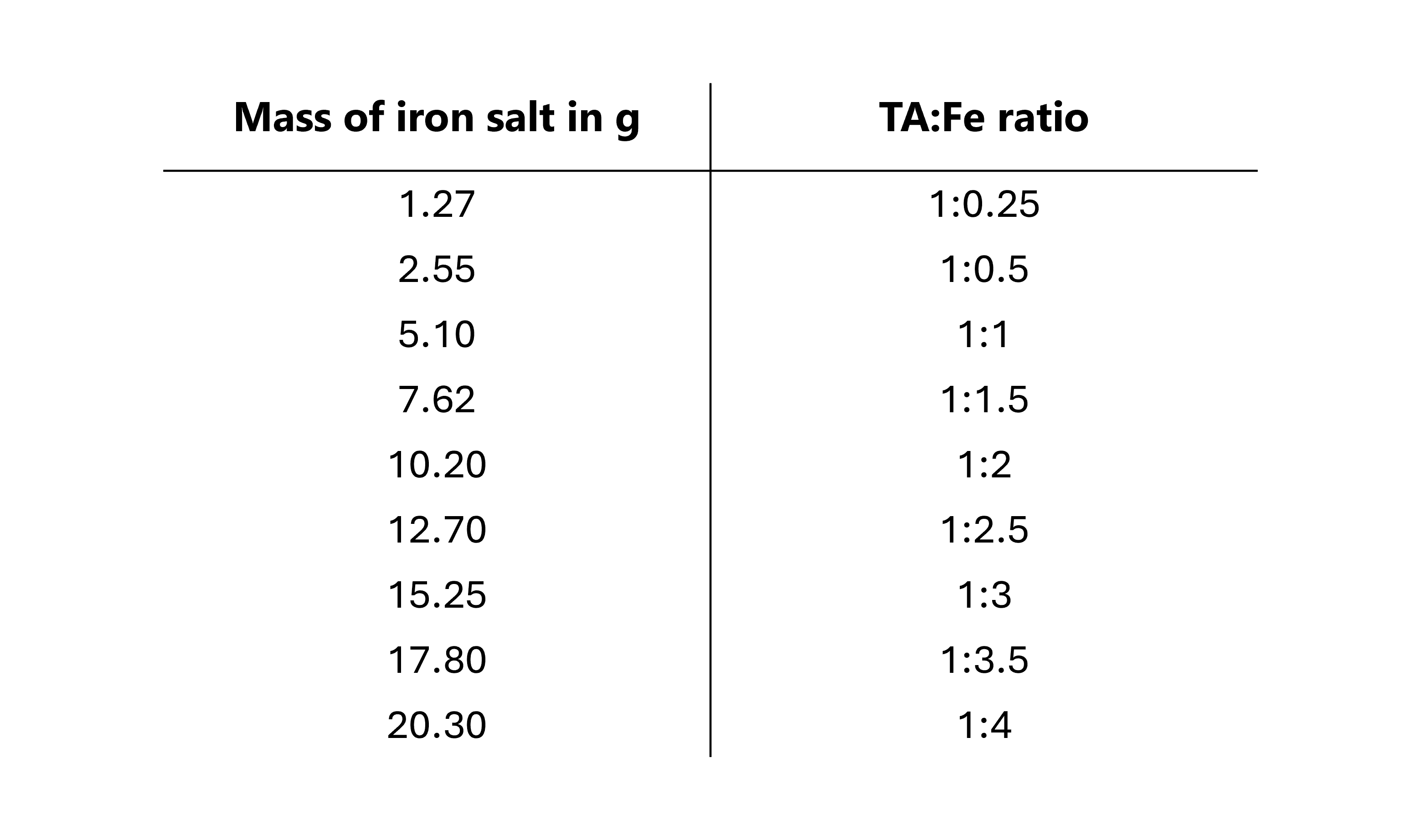
**

**
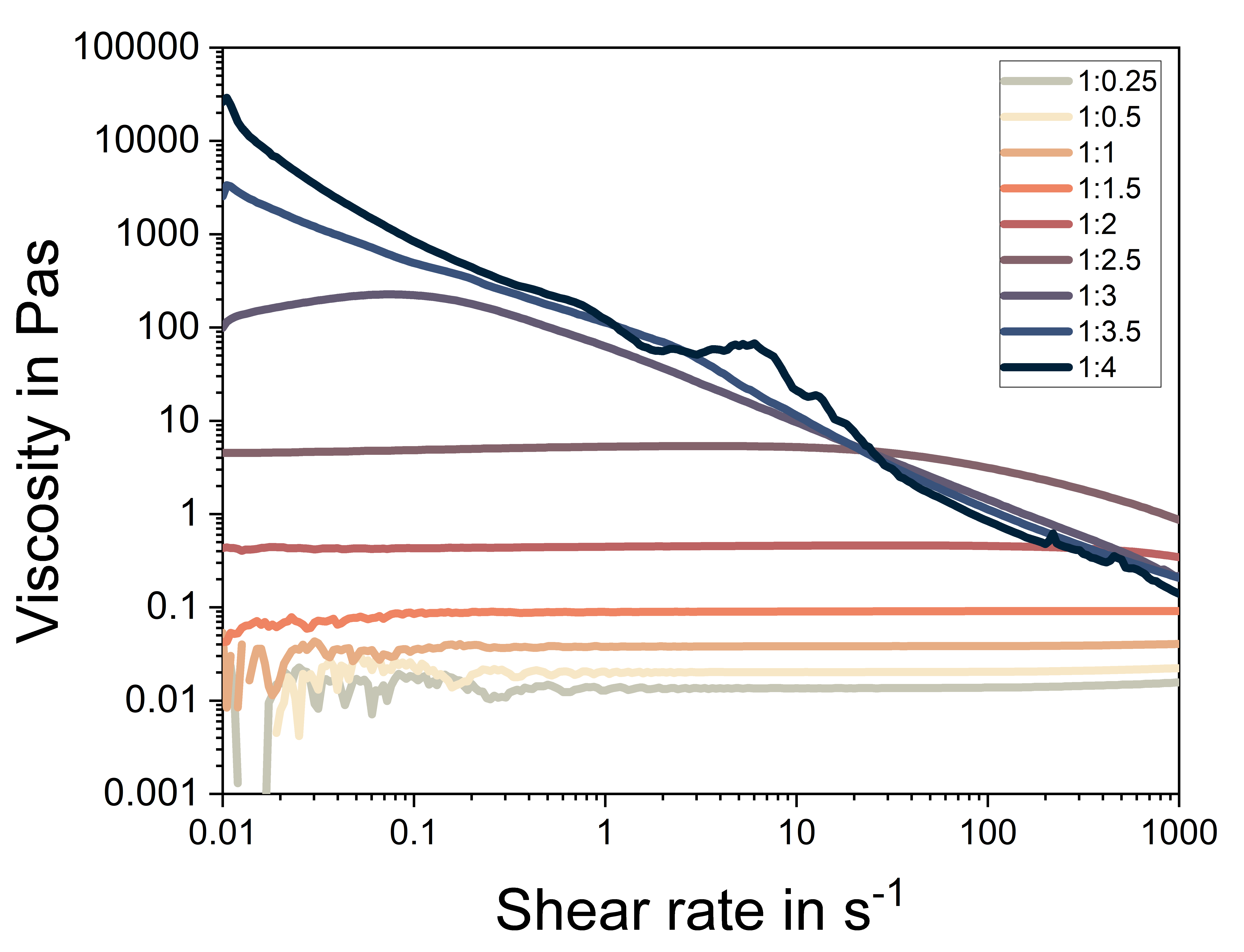
**

**Figure S1** Overview of the rheological properties of all inks highlighting the change in viscosity in dependence of the shear rate and the iron content.

**
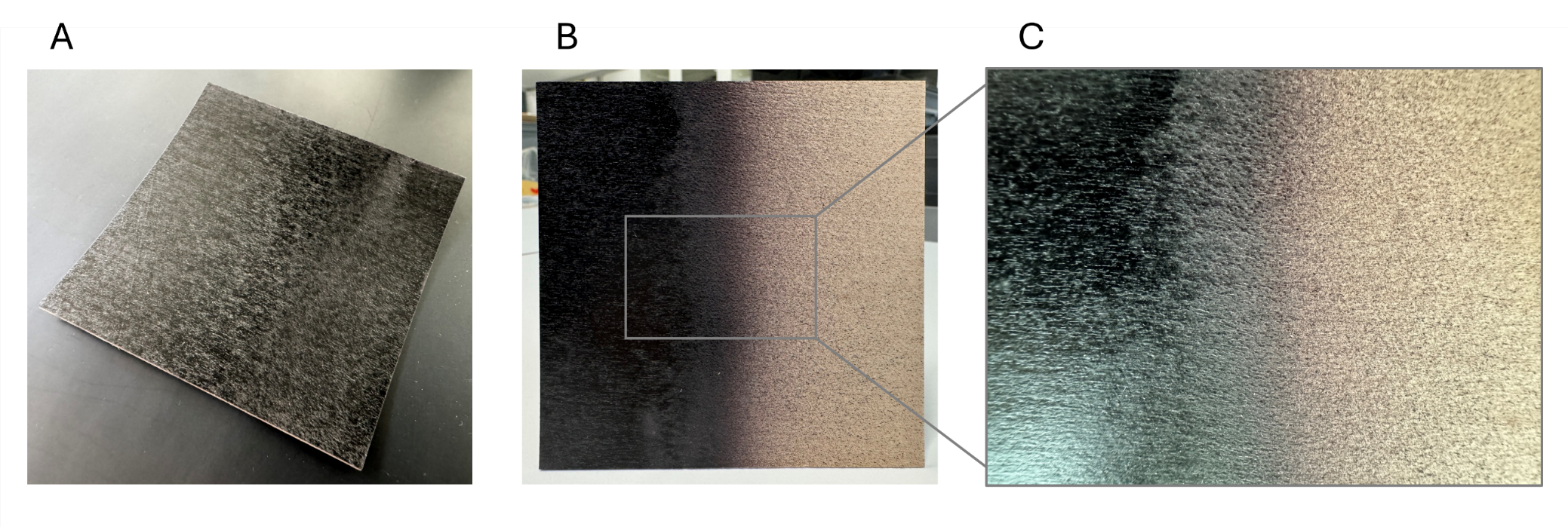
Figure S2** (A) shows a spray-coated maple wood veneer, highlighting the homogeneous and thin coating, with film thickness increasing upon the application of multiple layers and reaching approximately 50 µm after three consecutive layers. The progression from uncoated wood to one and two consecutive layers is shown in (B). Samples are 10 cm wide.

**
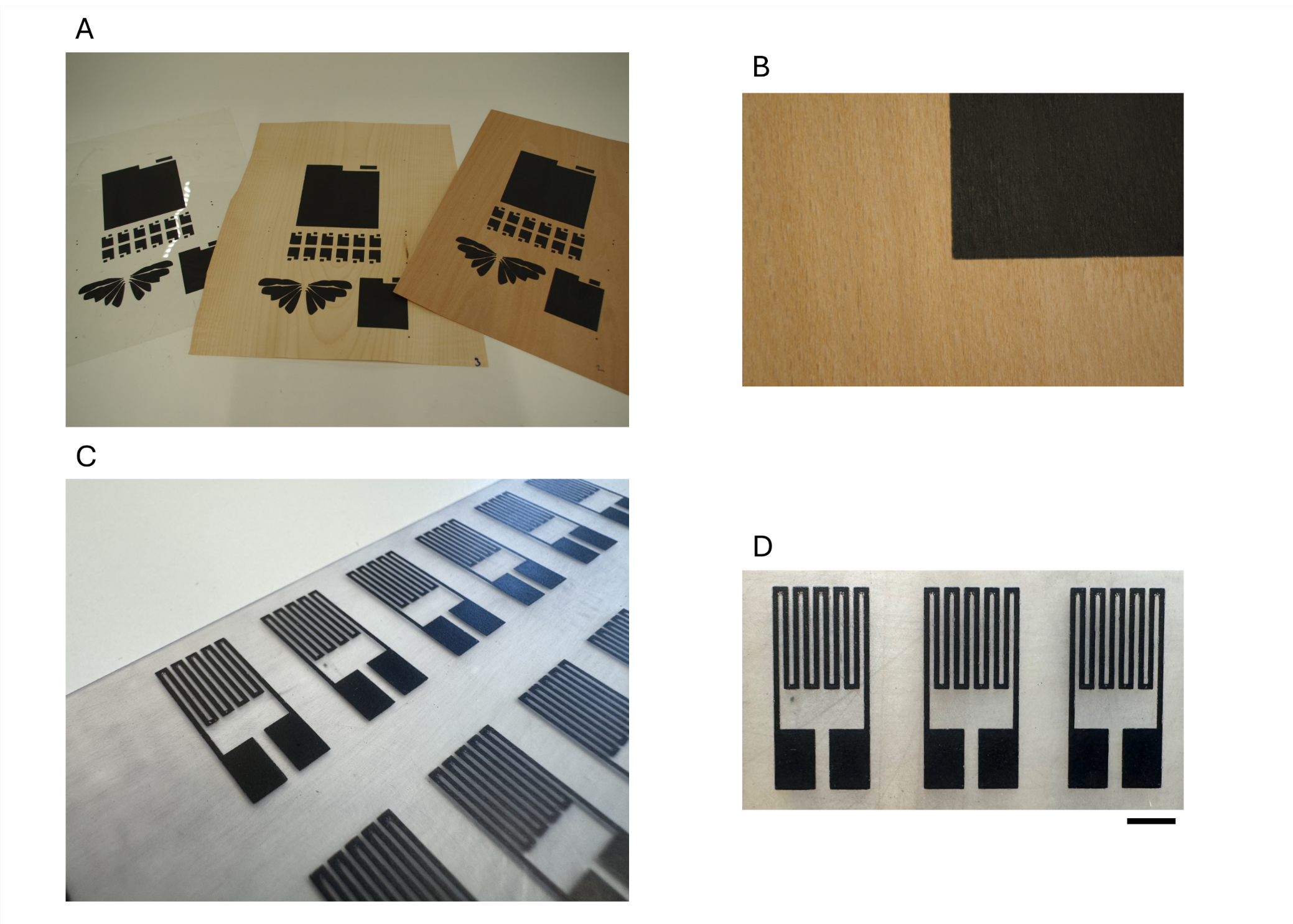
**

**Figure S3** (A) Diverse materials such as plastic foil (polyethylene terephthalate, PET) and different wood veneers (spruce left and beech right) can be used as substrates for semi-automated screen-printing showing minimal spreading (B), and precise details (C, D). Scale bar in (D) is 5mm.

**
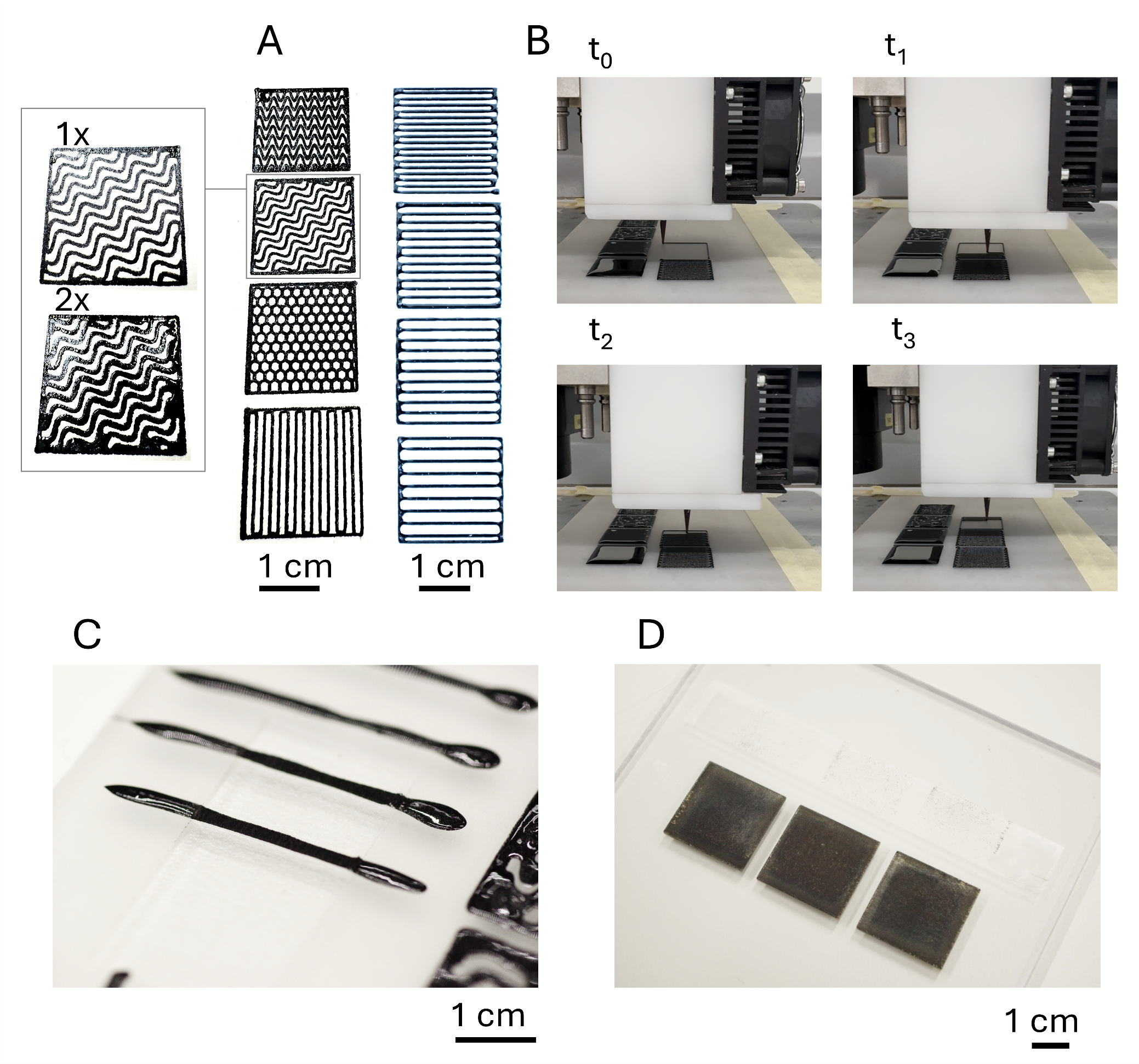
Figure S4** High-viscosity inks can be employed for the printing of complex patterns (A) using a 3D bioplotter (B). The inset in (A) shows one and two consecutively printed patterns.  (C) highlights the possibility of laser-treating a thick filament without ablation on a plastic (PET) substrate, while (D) highlights the minimal ablation observed after a qualitative peel test using Scotch tape on three IC-LIG electrodes (TA:Fe 1:3).

**Figure S5** Initial laser parameter study carried out with a TA:Fe ratio of 1:2, showing the electrical properties as a function of power and speed (A), allowing the IC-LIG to be used for various applications, such as an electrical resistor and conductor. The organic substrate used imposes constraints on the energy density that can be applied by laser treatment. Hence, high-power levels combined with low engraving speeds result in an irregular structure of the IC-LIG electrode, which may even lead to decomposition of the substrate (grey area; Figure S10). Refined parameter study (B) with a comparison of samples derived from different TA:Fe ratios, from 1:1 to 1:4.
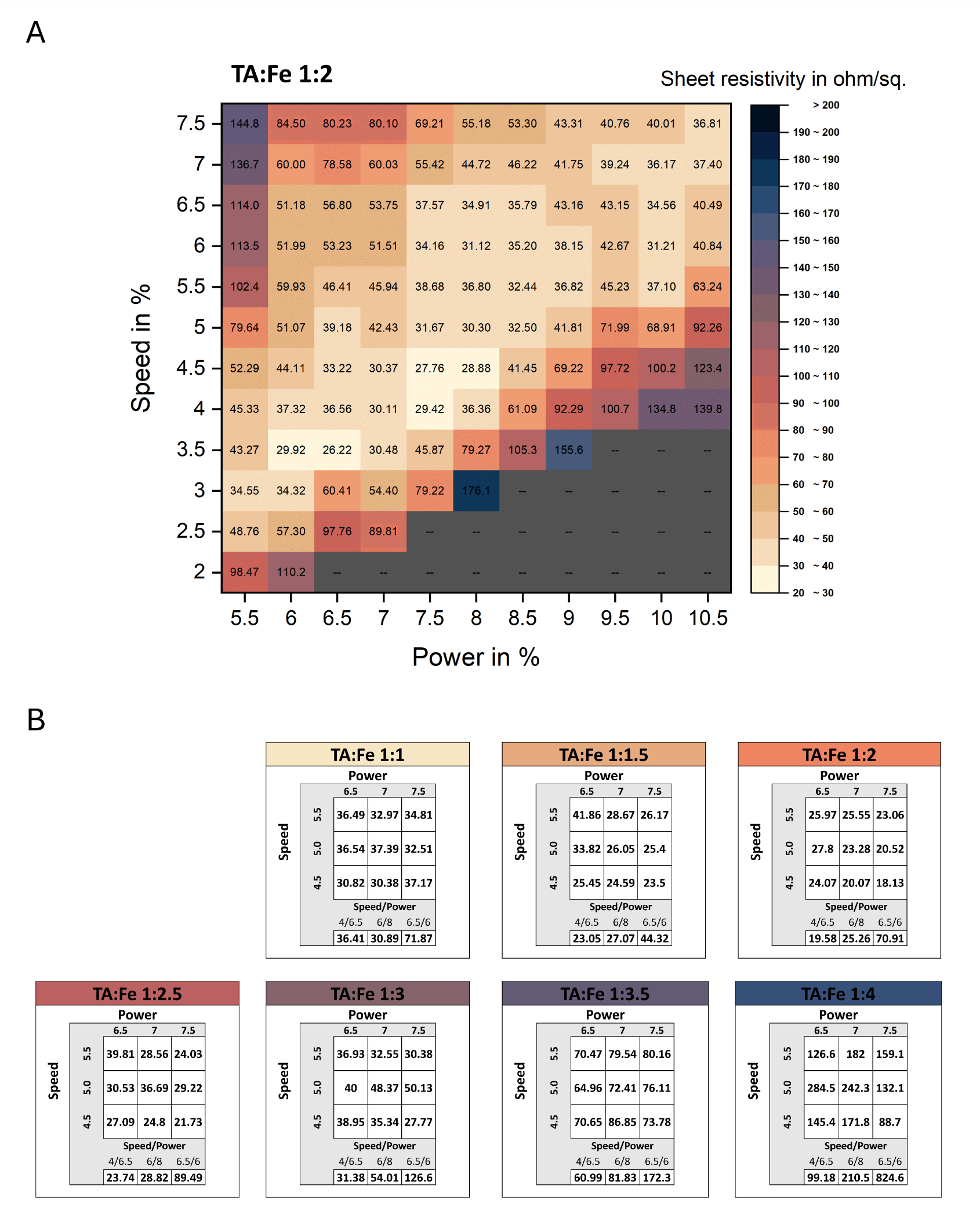


**Figure S6** (S)TEM analysis for TA:Fe ratios of 1:1 (A–H) and 1:3 (I–P): The highly graphitized top layer is shown in (A,B) for 1:1 and (I,J) for 1:3; SAED patterns with corresponding intensity profiles from rotational averaging are presented in (C,D) for 1:1 and (K,L) for 1:3. Iron-rich particles encapsulated in multilayer graphitic shells appear in (E,F) for 1:1 and (M,N) for 1:3, while oxygen-free particles are presented in (G,H) for 1:1 and (O,P) for 1:3.
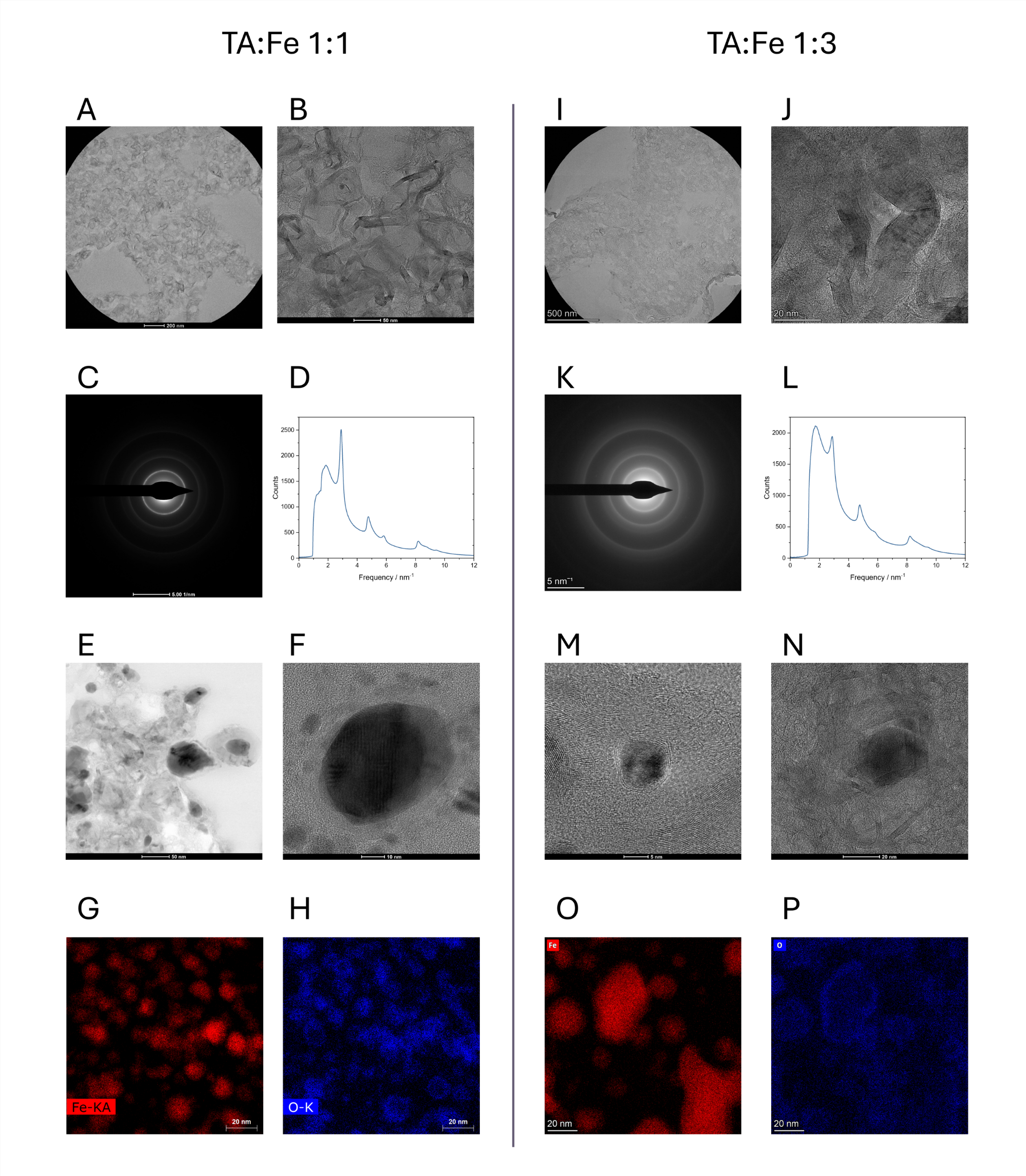


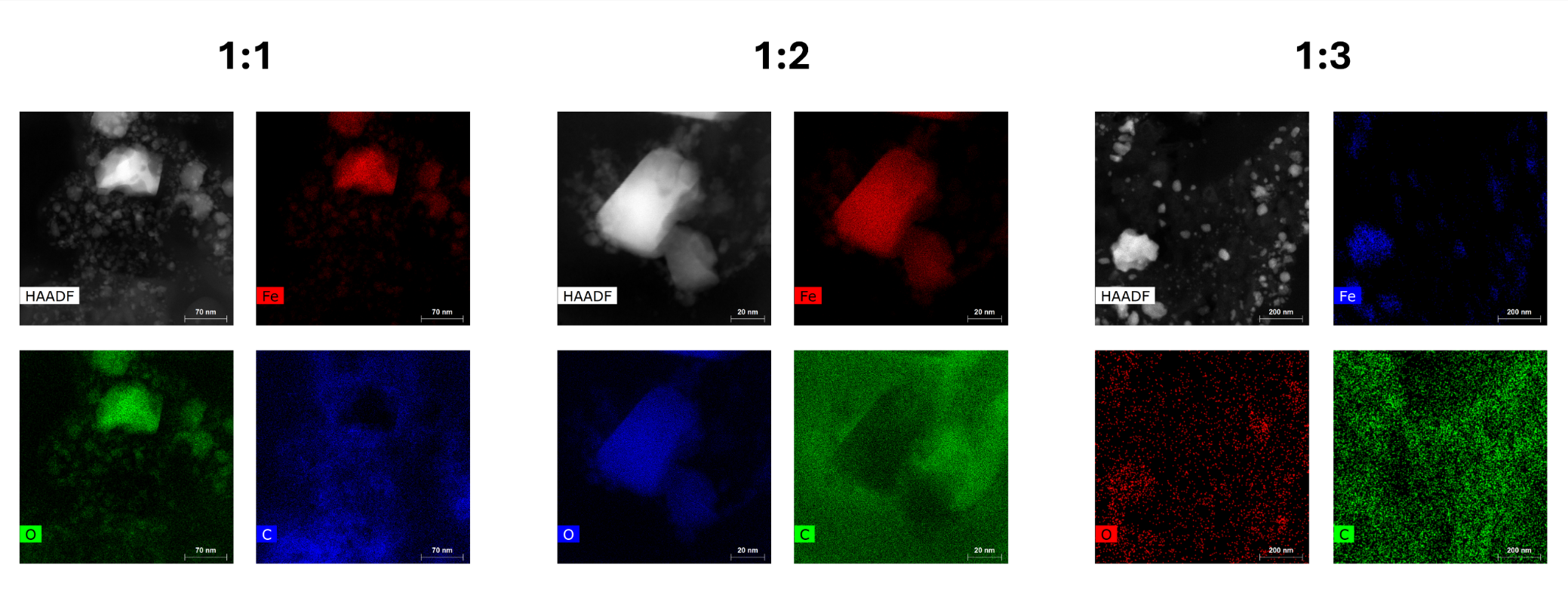


**Figure S7** Nanoparticles decorating the top layer of IC-LIG samples derived from TA:Fe ratios of 1:1, 1:2, and 1:3 were identified as magnetite particles.


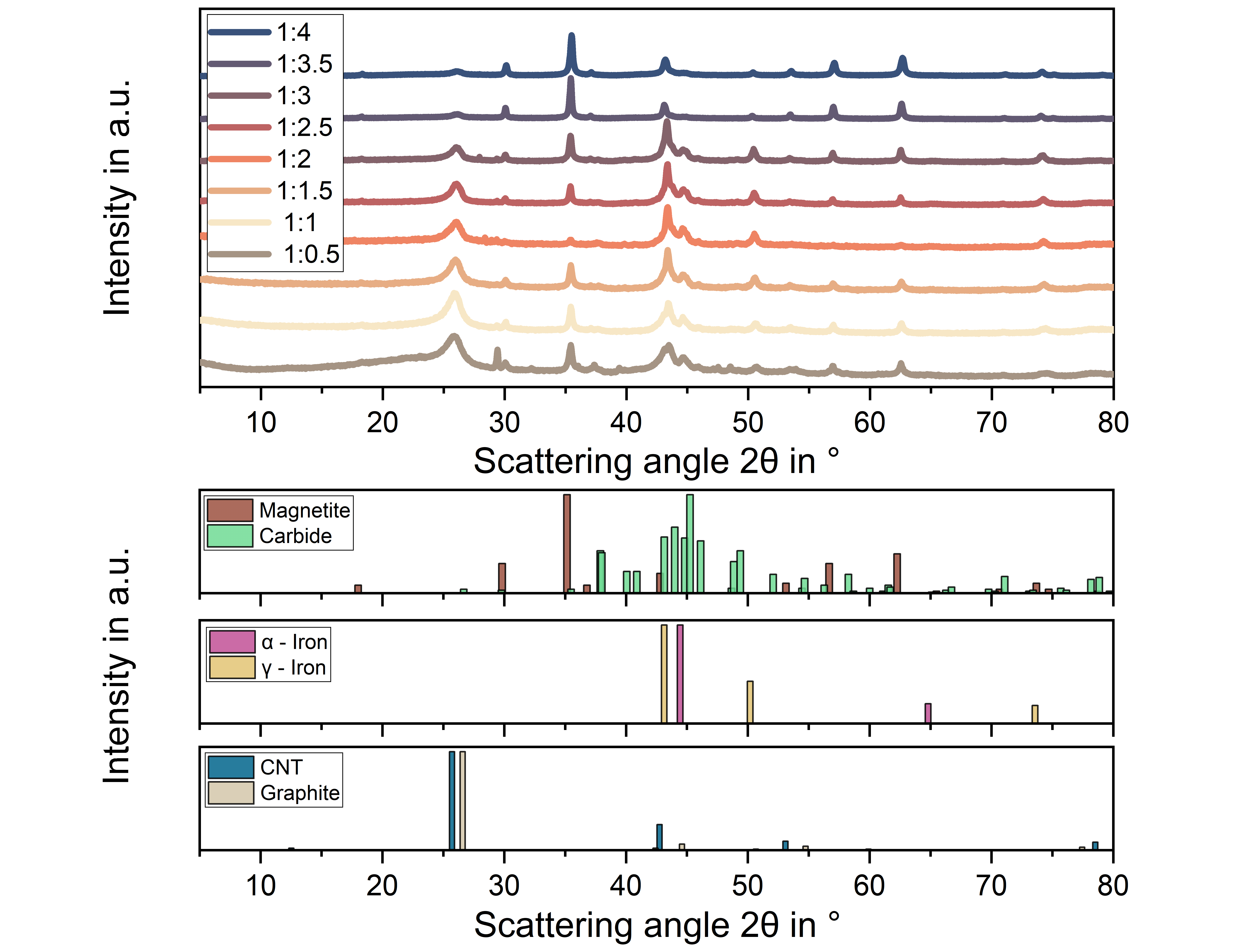
**Figure S8** Crystallographic analysis of all different TA:Fe ratios with corresponding reference spectra.


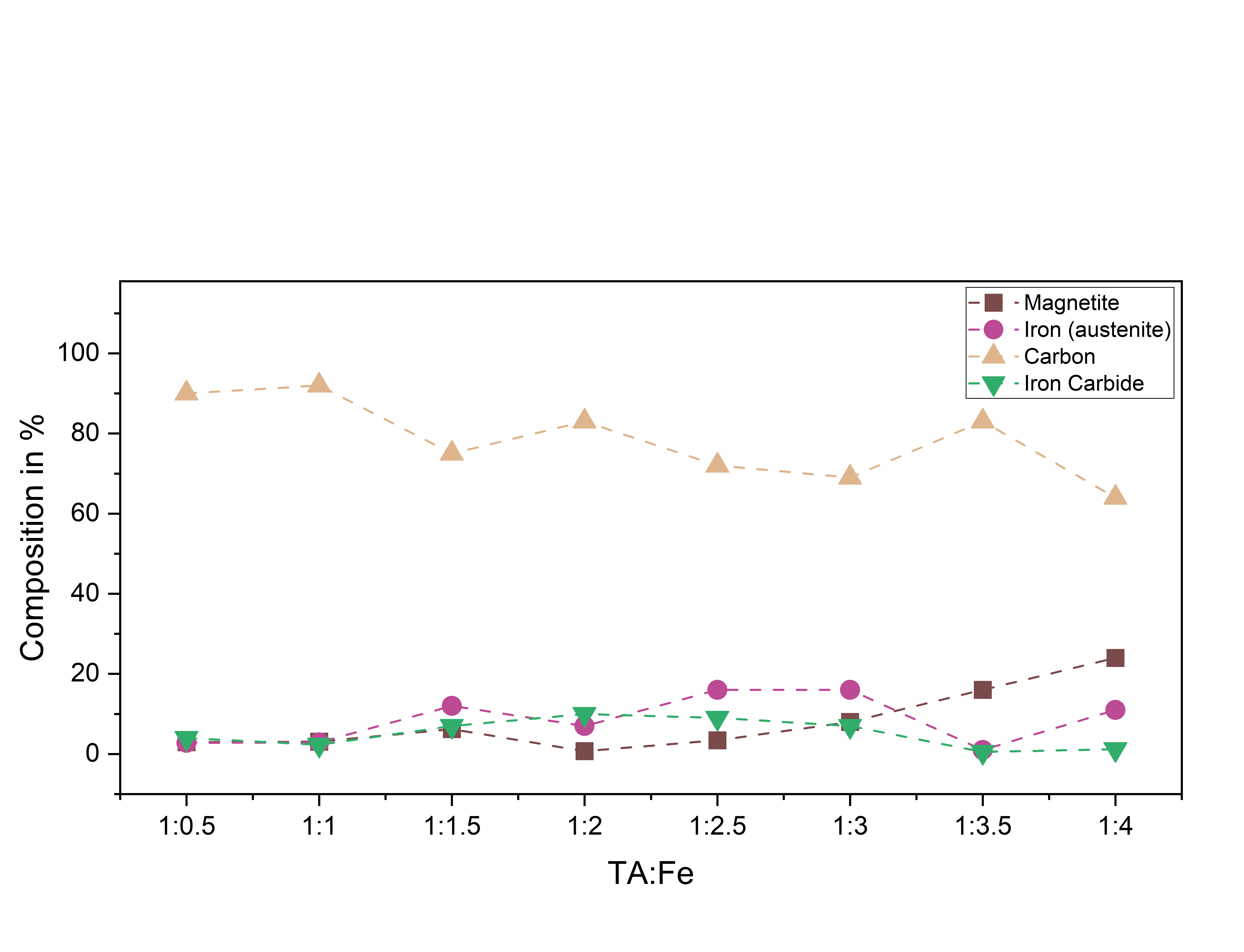


**Figure S9** Rietveld analysis of all investigated ink ratios, showing the corresponding phase composition, including iron species and carbon content.


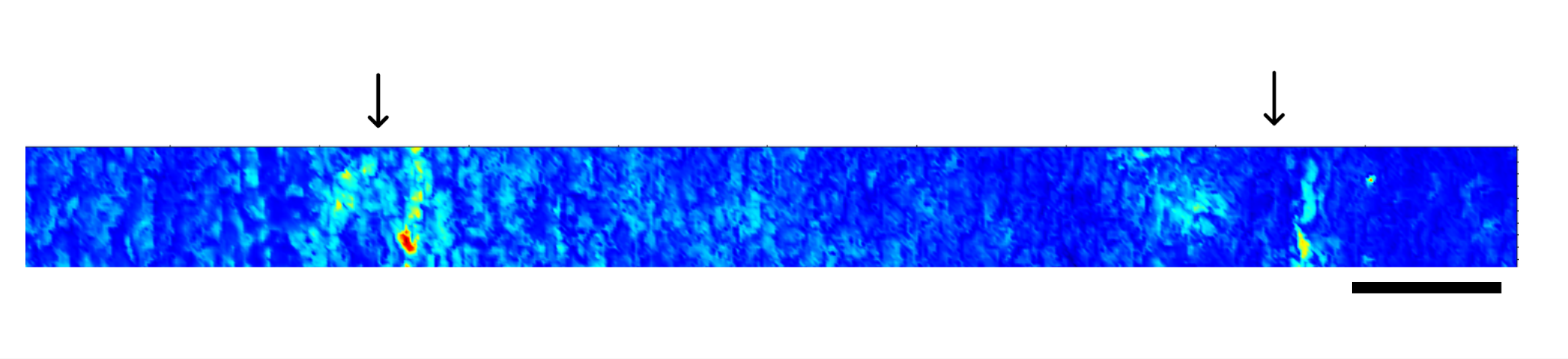


**Figure S10** Raman map (1450 to 1750 cm-1) with arrows indicating the double-lased areas, highlighting slight variations in the degree of graphitization. Scalebar is 250 µm.


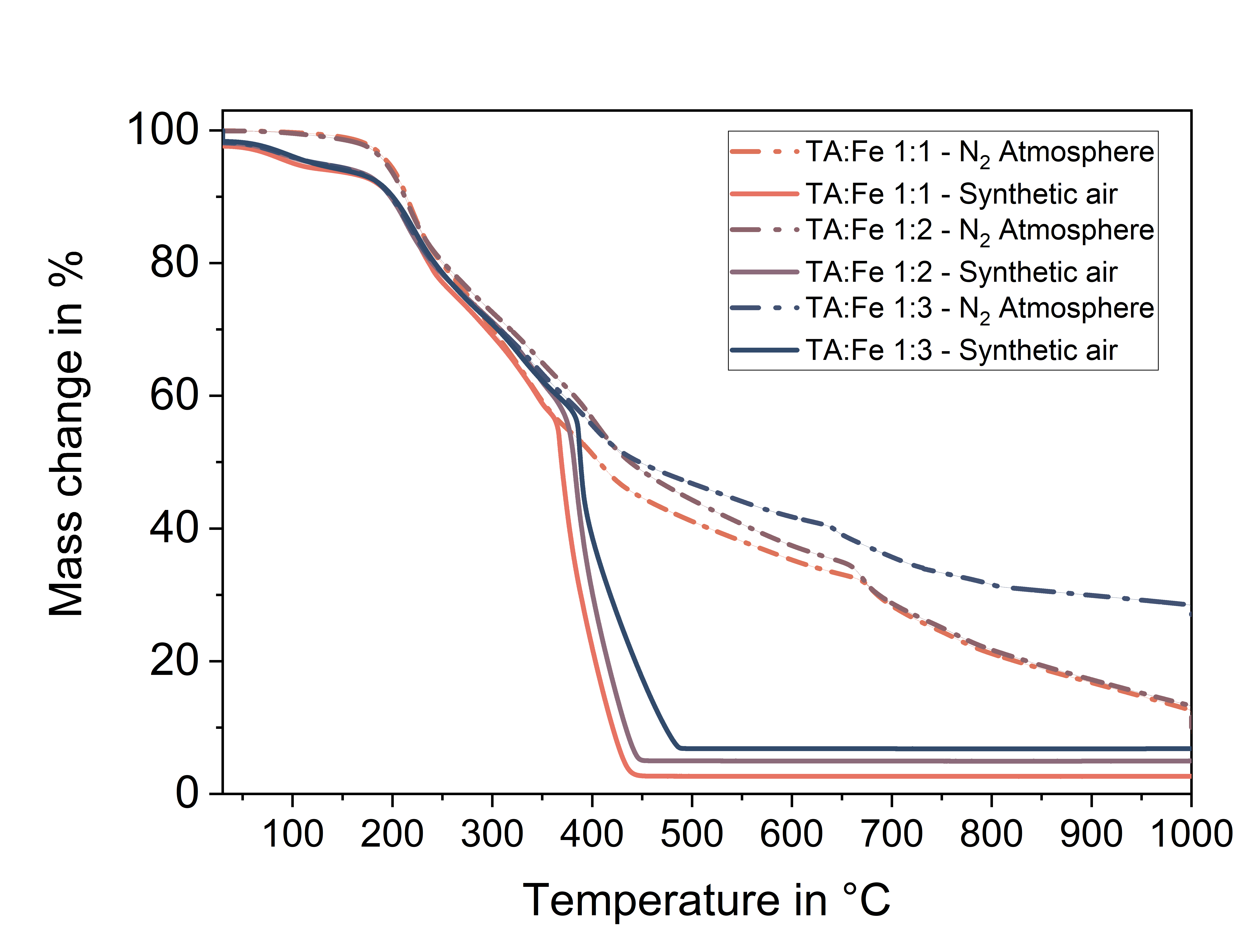


**Figure S11** Thermogravimetric analysis under inert nitrogen atmosphere and oxygen-rich synthetic air of three different TA:Fe ratios, 1:1, 1:2, and 1:3.


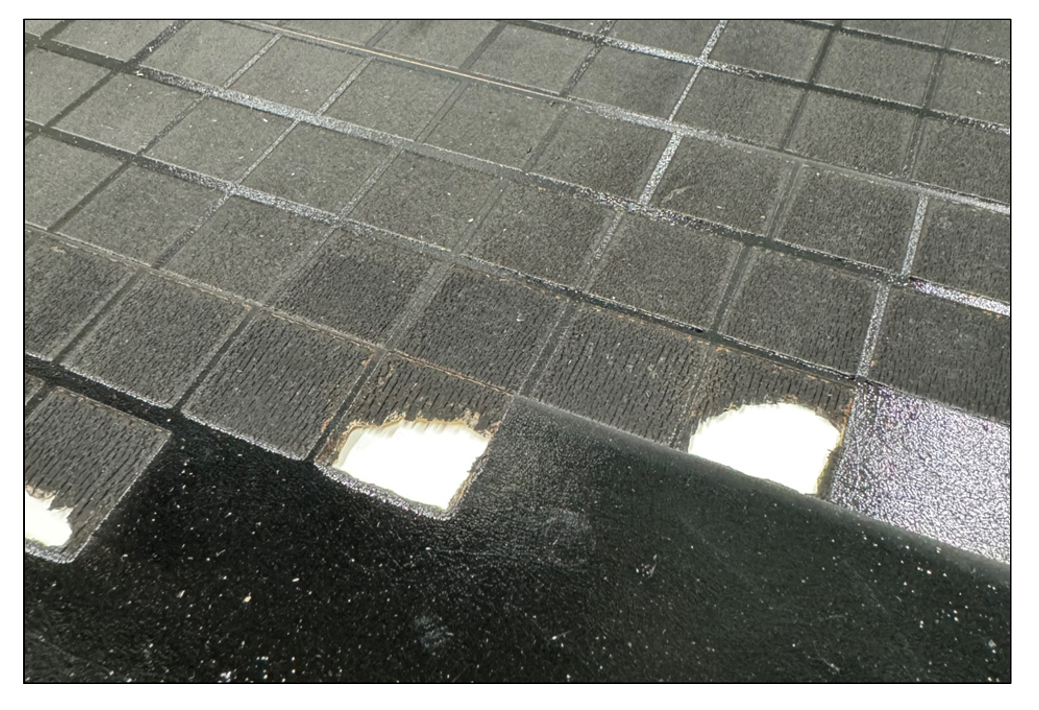


**Figure S12** Increased energy densities (high power, low speed: grey area of initial parameter study with IC-LIG samples derived from TA:Fe 1:2 ink) result in thermal damage and decomposition of the WPB substrate.


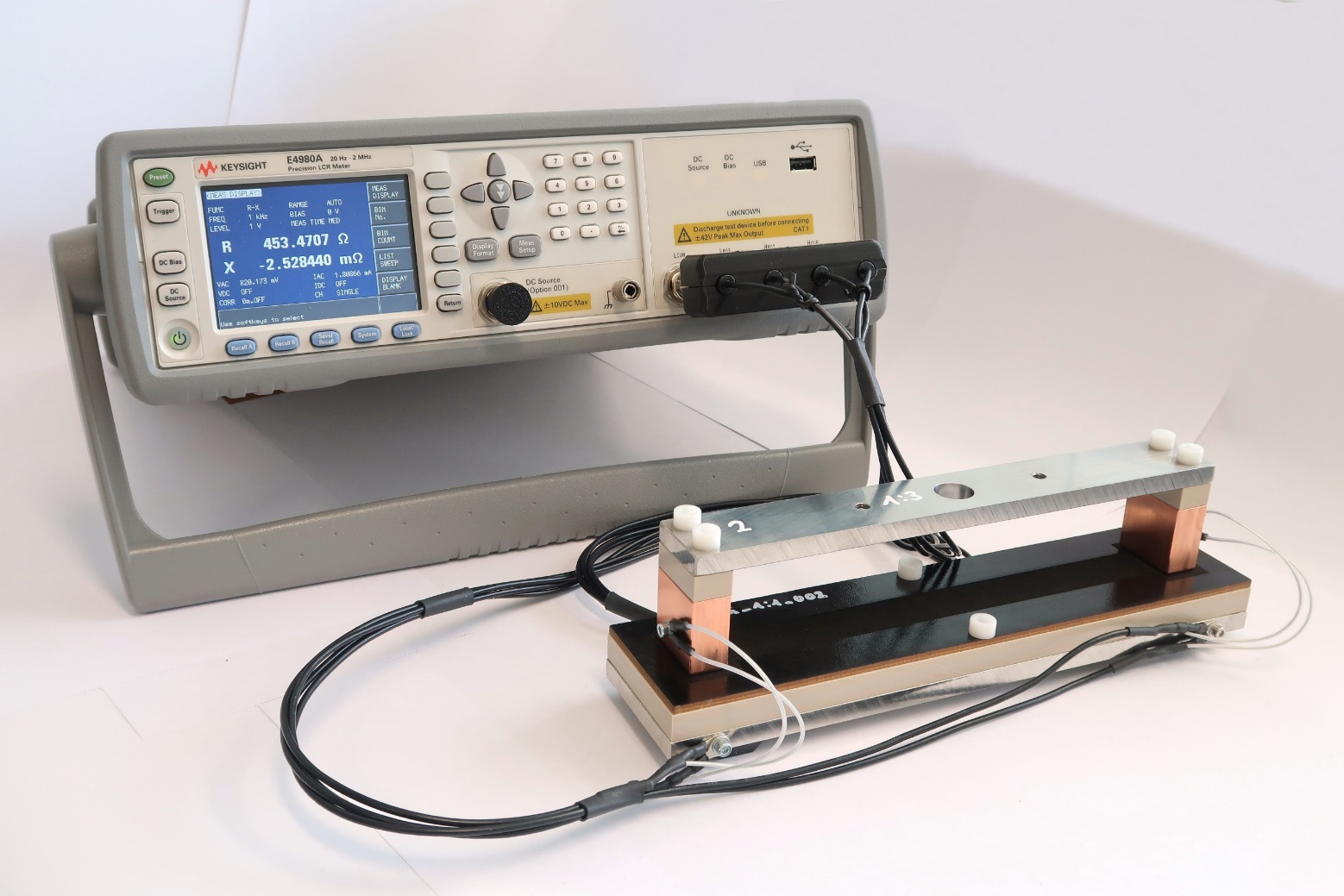

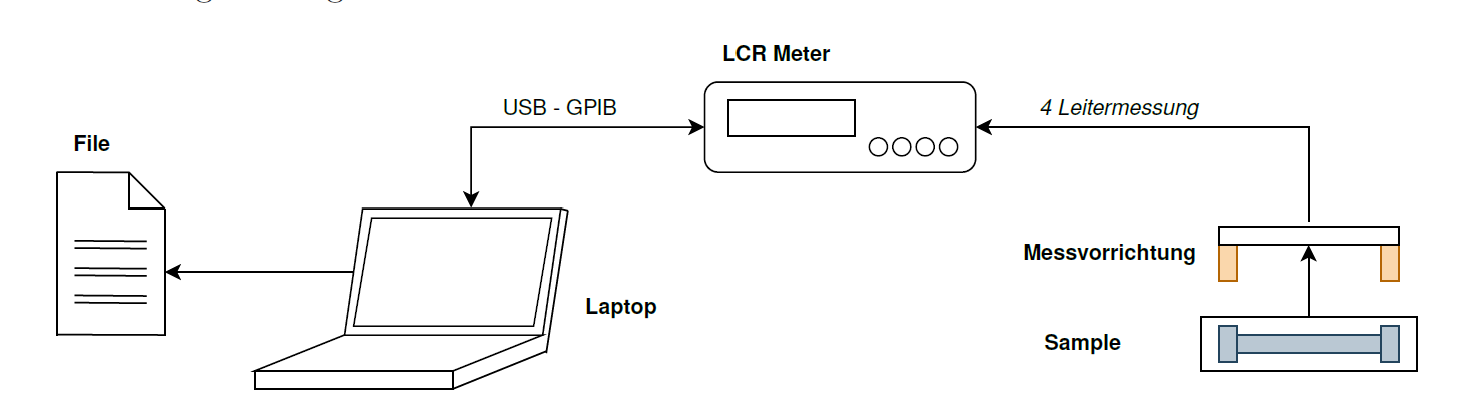


**Measurement setup**

**Figure S13** Schematic representation together with the test setup for the measurement of resistivity and reactance of IC-LIG electrodes.

**Figure S14** The dependency of the humidity and temperature on the reactance and resistivity is presented in A and B, respectively.


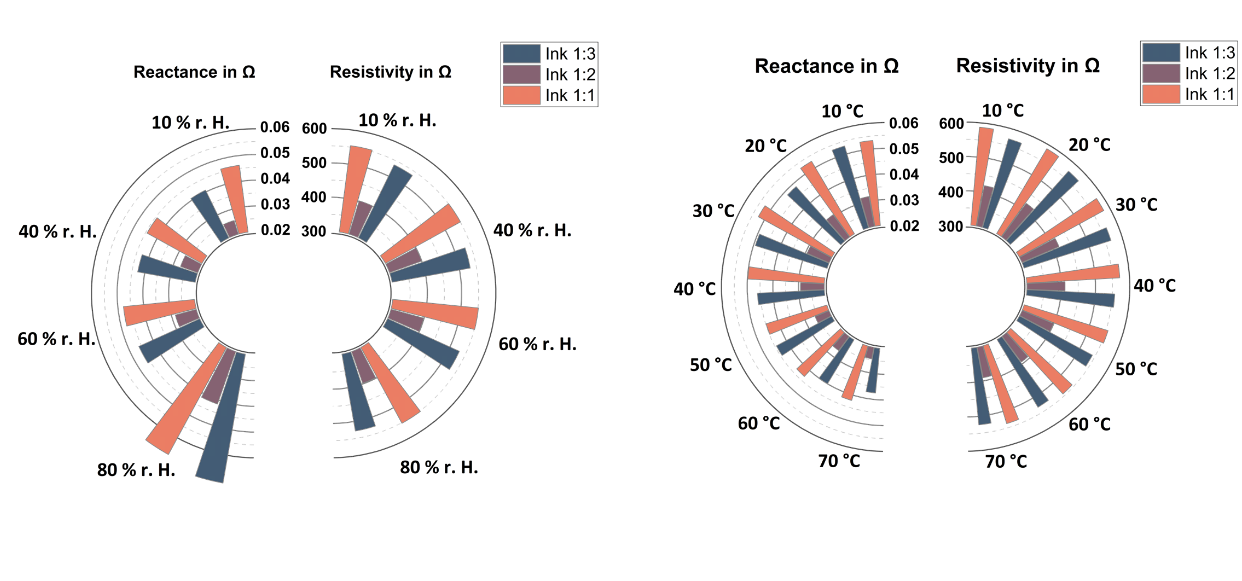


A

B


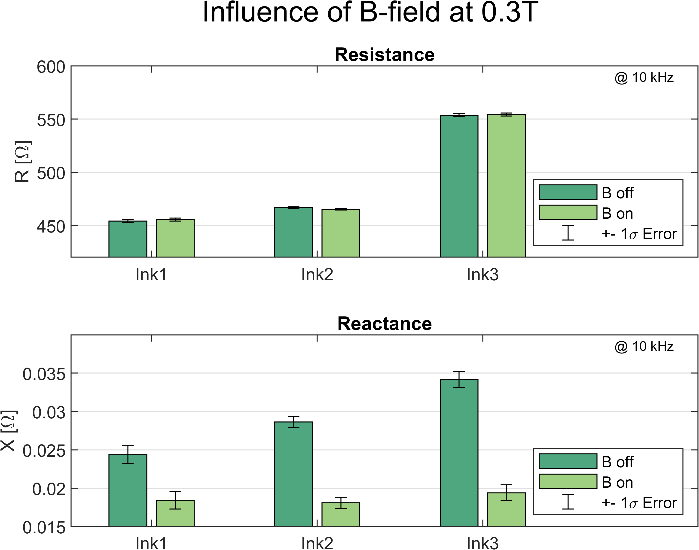

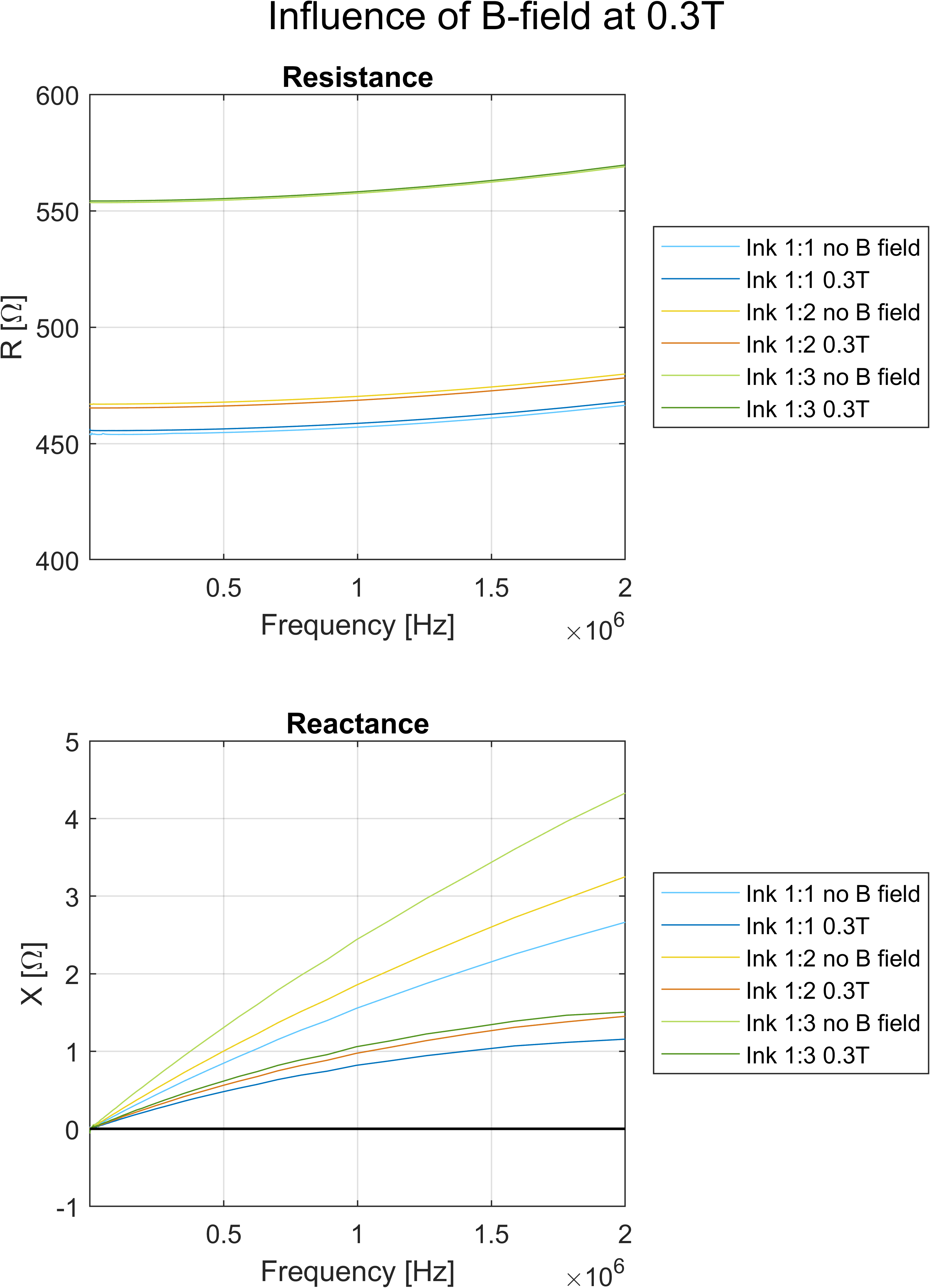

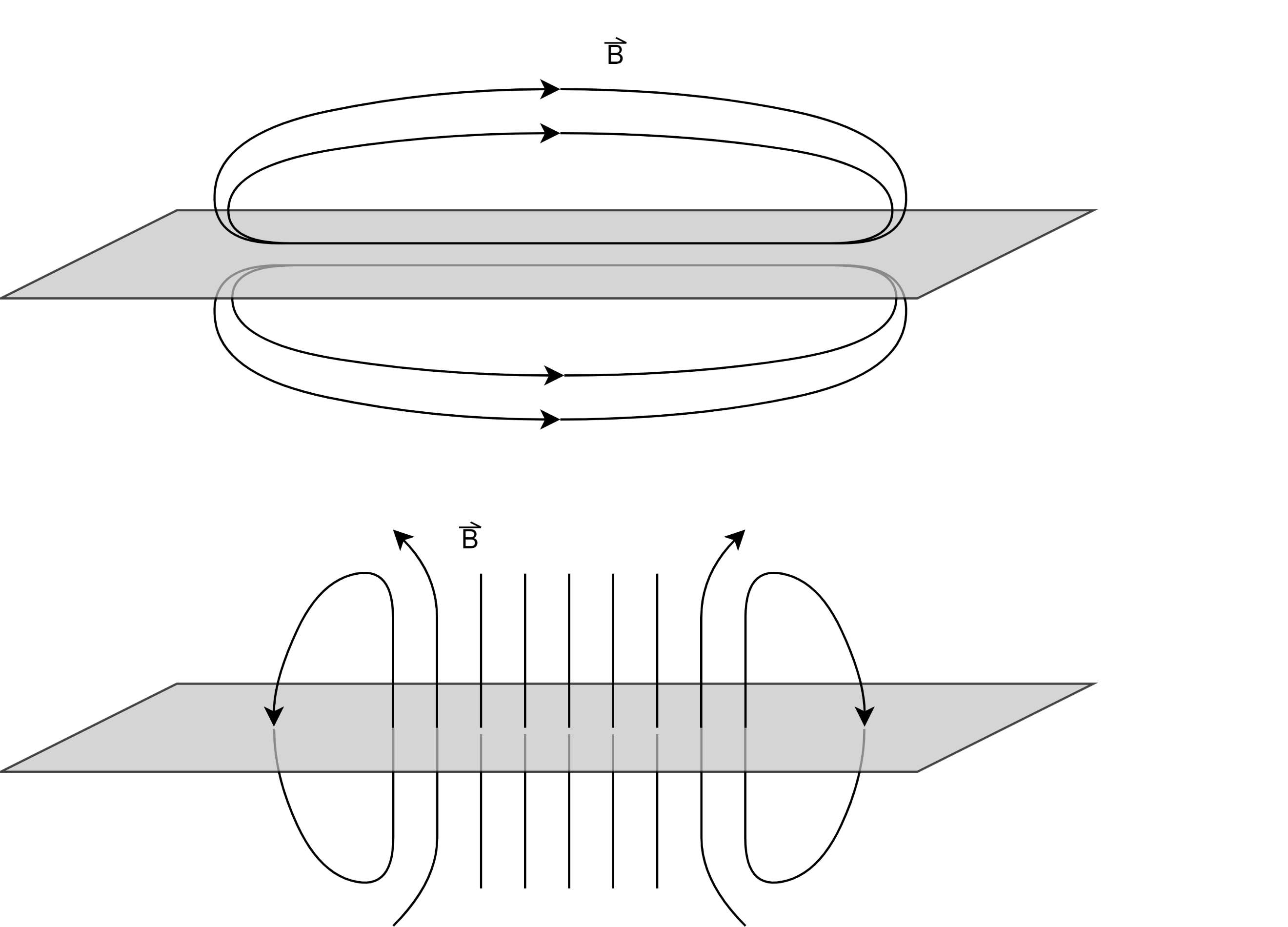


A

B

C

**Figure S15** Influence of the magnetic field (300mT) on the resistance and reactance in dependence of the TA:Fe ratio during the frequency sweep (A) and at constant frequency of 10kHz (C). The schematic representation depicts the measurement of a sample using neodymium permanent magnets (B), illustrating the magnetic field lines permeating through the surface normal of the sample during the measurement.


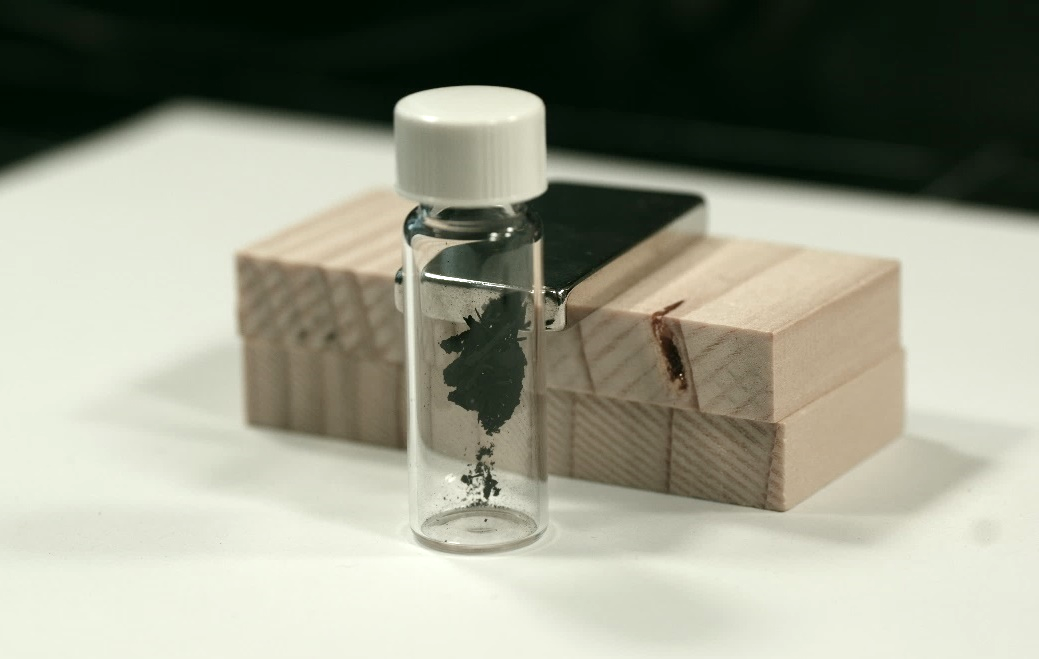


**Figure S16** Permanent magnet manipulates scratched IC-LIG powder flakes.


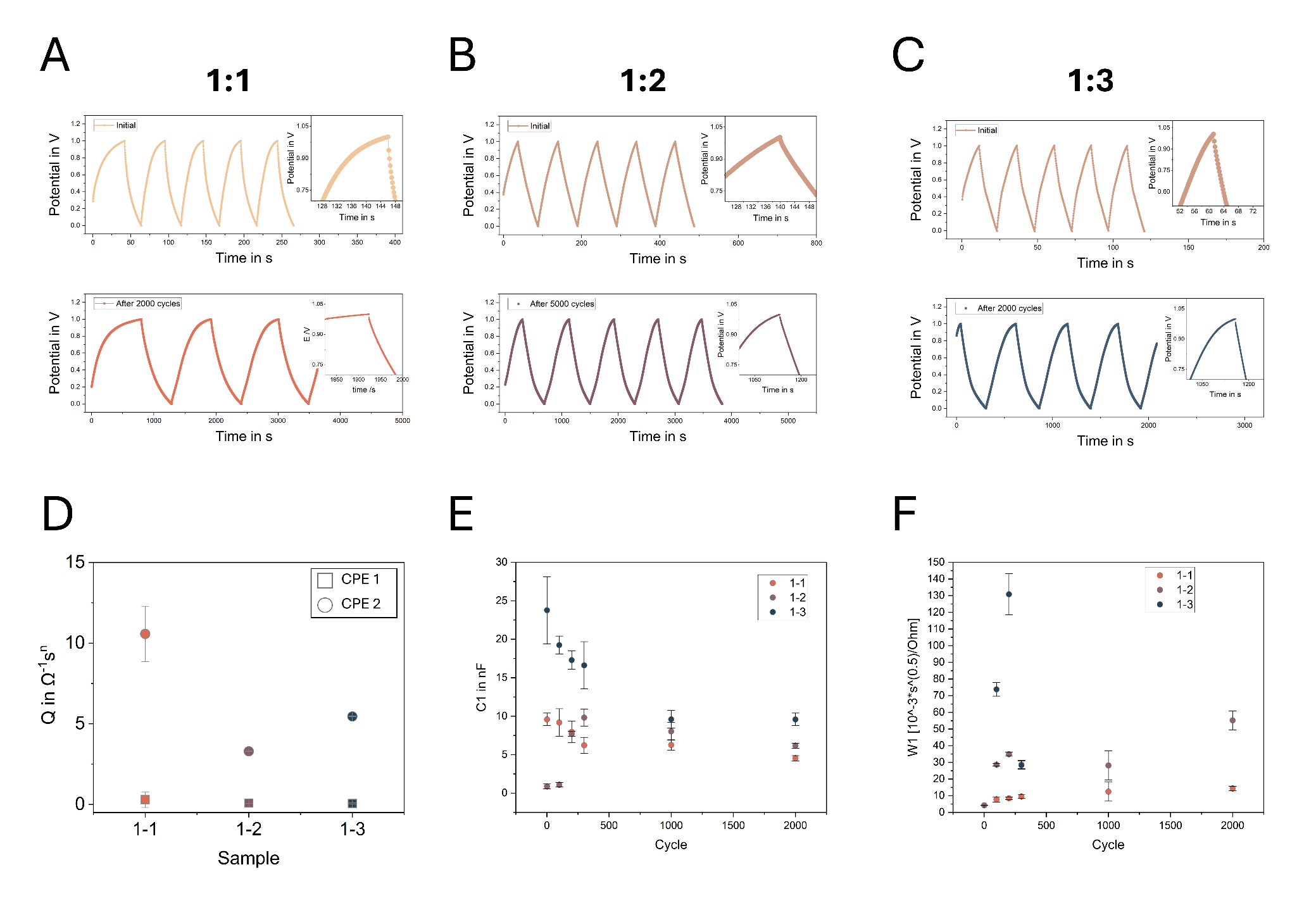


**Figure S17** Charge-discharge plots at 0.025 mA cm^-^² after 100 and 2000 cycles (5000 for sample 1:2) are presented in (A-C). Q_1_ Individual circuit elements of corresponding equivalent circuit model with results (CPE_1_), representing the surface capacitance (D), and the capacitor C1 (E) representing the cell capacity from the capacitance of the liquid medium as well as parasitic capacitances in dependency of the TA:Fe ratio and cycle number. The Warburg element showing an increase of its resistance which corresponds to limited ion penetration and increased tortuosity of the porous electrode (F).

**
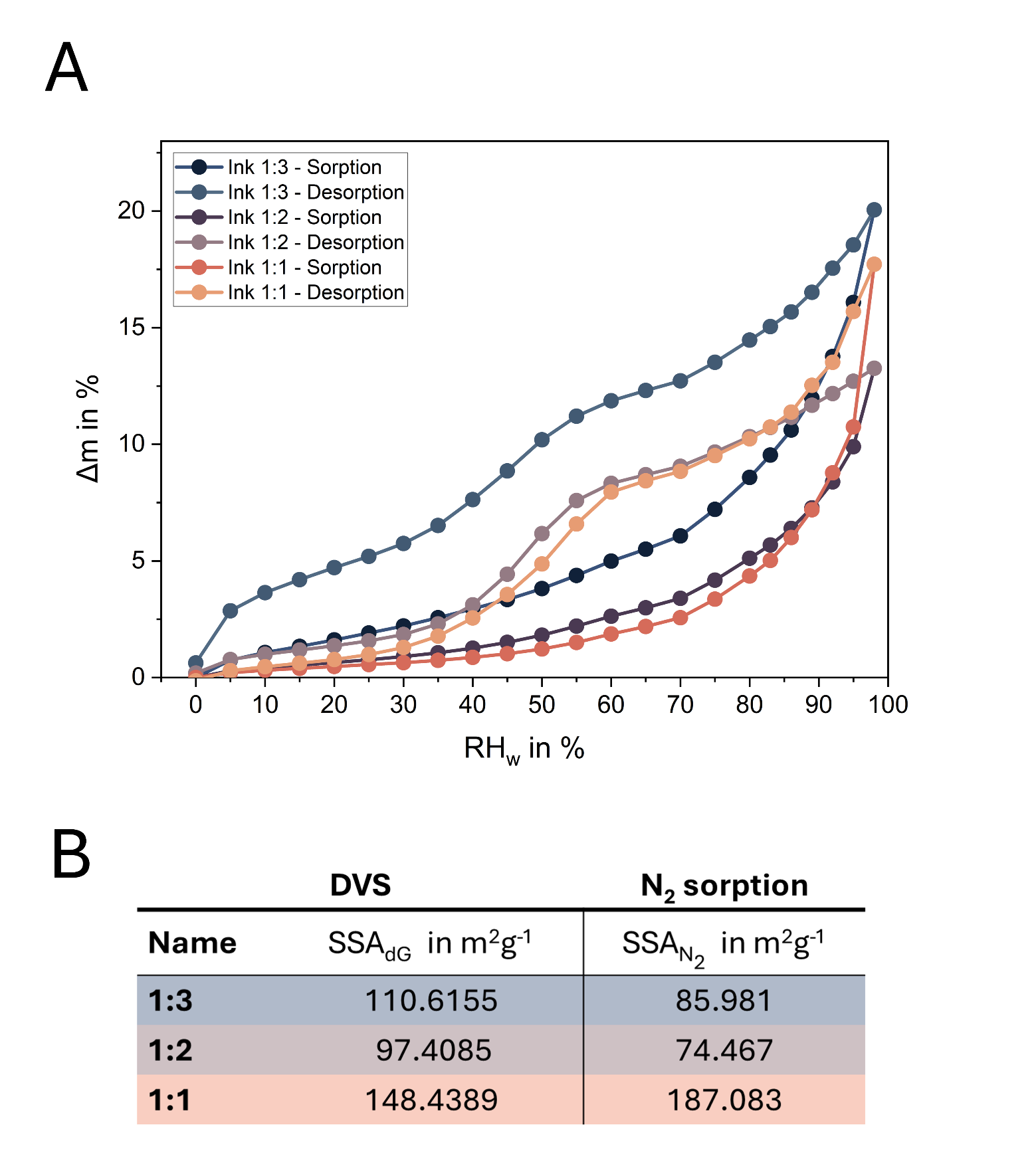
Figure S18** Dynamic vapor sorption (DVS) adsorption and desorption curves (B) with the corresponding estimation of the specific surface area derived from DVS and BET (N₂) sorption analysis.


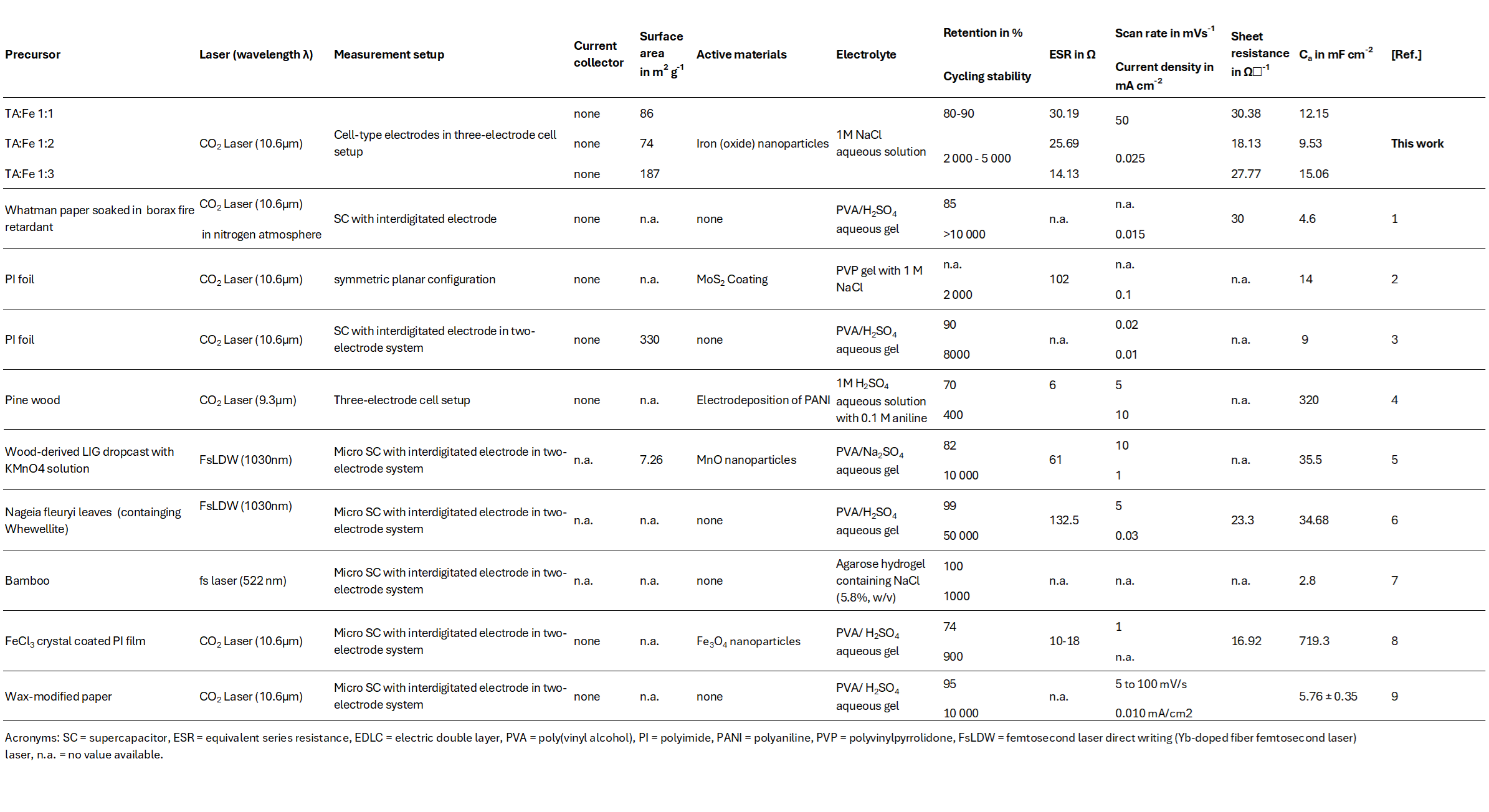


**Table S2** Key Performance Indicators (KPIs) of IC-LIG electrodes, including sheet resistance, electrochemical performance of three different TA:Fe ratios. The table includes a comparison to relevant literature values to contextualize the performance of the present study.

**References for Table S2**

[1] J. Coelho, R. F. Correia, S. Silvestre, T. Pinheiro, A. C. Marques, M. R. P. Correia, J. V. Pinto, E. Fortunato, R. Martins, Mikrochim Acta 2022, 190, 40.

[2] F. Clerici, M. Fontana, S. Bianco, M. Serrapede, F. Perrucci, S. Ferrero, E. Tresso, A. Lamberti, ACS Appl Mater Interfaces 2016, 8, 10459.

[3] Z. Peng, J. Lin, R. Ye, E. L. Samuel, J. M. Tour, ACS Appl Mater Interfaces 2015, 7, 3414.

[4] R. Ye, Y. Chyan, J. Zhang, Y. Li, X. Han, C. Kittrell, J. M. Tour, Adv Mater 2017, 29.

[5] Y.-R. Kim, H. K. Nam, Y. Lee, D. Yang, T.-S. D. Le, S.-W. Kim, S. Park, Y.-J. Kim, Biochar 2024, 6.

[6] T. S. D. Le, Y. A. Lee, H. K. Nam, K. Y. Jang, D. Yang, B. Kim, K. Yim, S. W. Kim, H. Yoon, Y. J. Kim, Advanced Functional Materials 2021, 32.

[7] R. Miyakoshi, S. Hayashi, M. Terakawa, RSC Adv 2022, 12, 29647.

[8] H. Liu, K.-s. Moon, J. Li, Y. Xie, J. Liu, Z. Sun, L. Lu, Y. Tang, C.-P. Wong, Nano Energy 2020, 77.

[9] T. Pinheiro, R. Correia, M. Morais, J. Coelho, E. Fortunato, M. G. F. Sales, A. C. Marques, R. Martins, ACS Nano 2022, 16, 20633.
